# Metabolic peculiarities of *Aspergillus niger *disclosed by comparative metabolic genomics

**DOI:** 10.1186/gb-2007-8-9-r182

**Published:** 2007-09-04

**Authors:** Jibin Sun, Xin Lu, Ursula Rinas, An Ping Zeng

**Affiliations:** 1 Helmholtz Centre for Infection Research, Inhoffenstr., 38124 Braunschweig, Germany; 2 Hamburg University of Technology, Institute of Bioprocess and Biosystems Engineering, Denickestr., 21071 Hamburg, Germany

## Abstract

**Background:**

*Aspergillus niger *is an important industrial microorganism for the production of both metabolites, such as citric acid, and proteins, such as fungal enzymes or heterologous proteins. Despite its extensive industrial applications, the genetic inventory of this fungus is only partially understood. The recently released genome sequence opens a new horizon for both scientific studies and biotechnological applications.

**Results:**

Here, we present the first genome-scale metabolic network for *A. niger *and an in-depth genomic comparison of this species to seven other fungi to disclose its metabolic peculiarities. The raw genomic sequences of *A. niger *ATCC 9029 were first annotated. The reconstructed metabolic network is based on the annotation of two *A. niger *genomes, CBS 513.88 and ATCC 9029, including enzymes with 988 unique EC numbers, 2,443 reactions and 2,349 metabolites. More than 1,100 enzyme-coding genes are unique to *A. niger *in comparison to the other seven fungi. For example, we identified additional copies of genes such as those encoding alternative mitochondrial oxidoreductase and citrate synthase in *A. niger*, which might contribute to the high citric acid production efficiency of this species. Moreover, nine genes were identified as encoding enzymes with EC numbers exclusively found in *A. niger*, mostly involved in the biosynthesis of complex secondary metabolites and degradation of aromatic compounds.

**Conclusion:**

The genome-level reconstruction of the metabolic network and genome-based metabolic comparison disclose peculiarities of *A. niger *highly relevant to its biotechnological applications and should contribute to future rational metabolic design and systems biology studies of this black mold and related species.

## Abstract 2

A genome-scale metabolic network and an in-depth genomic comparison of *Aspergillus niger *with seven other fungi is presented, revealing more than 1,100 enzyme-coding genes that are unique to *A. niger*.

## Background

Metabolic network reconstruction based on the knowledge of annotated genomic sequences is a prerequisite to fully understand and exploit the metabolic potential of industrially relevant organisms. Modern fast DNA-sequencing methods as well as state-of-the-art bioinformatic tools are nowadays available for the reconstruction and cross-comparison of these networks among related species as well as among specific strains in order to elucidate their metabolic peculiarities.

Among the filamentous genus *Aspergillus*, *A. niger*, *A. awamori *(a subspecies of *A. niger*) and *A. oryzae *are the industrially important producers of both metabolites and enzymes [1]. For example, citric acid is nowadays almost exclusively produced using *A. niger*, although this "well-working black box" is not yet fully understood [2]. In addition, *A. niger *has also revealed some potential in bioremediation [3-6] and, moreover, it is a well-known producer of extracellular fungal enzymes. For example, glucoamylase in 20 grams per liter quantities have been reported [7]. Based on these secretion capacities, many efforts have also been undertaken to develop *A. niger *as a producer of heterologous proteins such as biopharmaceuticals [8,9], most often with limited success. Thus, there is a great need for a better knowledge of the genomic potential of *A. niger*, which could be used for rational strain improvement.

By now, the full genomes of *A. nidulans *[10], *A. oryzae *[11], and the human pathogen *A. fumigatus *[12] have been determined. Compared to *A. nidulans*, which has been widely used as the model organism for studies on fungal physiology and genetics, very little is known about the genetic background of *A. niger*. Only recently, the annotated genomic sequence of *A. niger *became publicly available [13], now allowing a more in-depth analysis of the metabolic potential of this important black fungus as well as the application of modern 'omics' technologies to further improve its performance.

A small-scale metabolic network can be reconstructed based on experimental evidence derived from the literature. However, reconstruction of a more complete, or so-called genome-scale, metabolic network relies on having the genome sequence and high-quality genome annotation [14,15]. Briefly, a list of enzymes, especially Enzyme Commission (EC) numbers, is extracted from the genome annotation and searched in an established biochemical reaction database to acquire their corresponding reactions. The biochemical reactions are then connected to each other according to certain rules [15]. Such information can be further interpreted as a network and analyzed by many computer programs, such as Cytoscape [16].

The model of *A. niger *central metabolism was reported previously [13,17]. In this study, we reconstructed a genome-scale metabolic network from the annotated genome of *A. niger *CBS 513.88 [13]. Moreover, from raw genomic data (three-fold coverage) of *A. niger *ATCC 9029 (Integrated Genomics, Chicago, IL, USA), protein coding sequences (CDSs) were identified, annotated and used for a more complete metabolic network reconstruction. The high-resolution *A. niger *metabolic network was cross-compared between the two *A. niger *strains as well as to other *Aspergillus *species (*A. nidulans*, *A. oryzae*, *A. fumigatus*, *A. flavus*) and other filamentous fungi, such as *Fusarium graminearum *and *Neurospora crassa*, and to the yeast *Saccharomyces cerevisiae *for identification of unique genes and metabolic peculiarities. Finally, selected genes from the citric acid production pathway of *A. niger *CBS 513.88 and *A. niger *ATCC 9029 were cross-compared to the respective genes of *A. niger *ATCC 1015, whose genome was recently released by the Joint Genomics Institute ahead of annotation [18].

## Results and discussion

### Genomic annotation of the low-coverage genome of *A. niger *ATCC 9029

**Figure 1:**
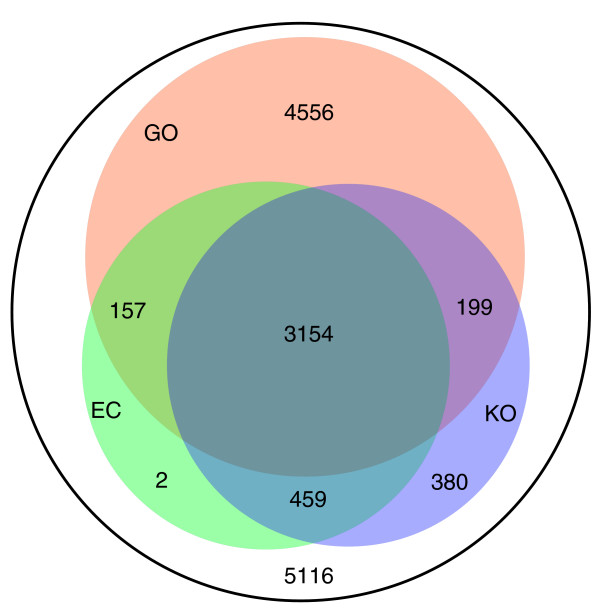
Functional annotation of predicted protein coding sequences of *A. niger*.

The unannotated raw genome sequence of *A. niger *ATCC 9029 from Integrated Genomics was annotated by using an improved version of the program 'IdentiCS' (see Materials and methods and Additional data file 1) with a cutoff E-value of 1E-5. The combination of results from the algorithms IdentiCS, GeneWise [19] and GenScan [20] resulted in a protein database of *A. niger *with approximately 16,000 entries. Of these, 75% are located on the ends of contigs, obviously because of the small size of the contigs due to a low genomic coverage of the sequence and the larger size of genes due to the presence of introns. Nearly 4,000 coding sequences (CDSs) were merged into about 2,000 entries using homologous protein sequences from the NCBI database as hints by a method described previously [21]. The final *A. niger *protein database contains 14,023 entries. By applying the strategies described in the Materials and methods section, the annotation was improved to address the functionality of the coding sequences in terms of Gene Ontology (GO), KEGG orthology (KO), Clusters of Orthologous Groups (COG), EC numbers, pathways, and so on. Two-thirds of the identified CDSs were assigned to at least one functional category (Figure 1); 8,066 CDSs were assigned to the GO category, 4,192 to the KO/COG category and 3,772 to EC numbers.

### Comparative genomics

To understand the unique genetic makeup of *A. niger *that accounts for its high capacity in various industrial processes, the annotated CDSs of *A. niger *ATCC 9029 from this study and CBS 513.88 from the Dutch company DSM [13] were cross-compared with seven selected fungi for which genome data are available. Based on the 15,720 ortholog groups established by the program OrthoMCL [22] (see Additional data file 2 for a complete list of the orthologs in the compared organisms), we show the pairwise comparison of the proteomes in Table 1. If the ortholog of a gene from one organism is absent in another organism, we define that this gene is unique or specific to the first organism in comparison to the second one (see Materials and methods for details). There exist remarkable differences among the fungi compared, even in the genus *Aspergillus*. Up to 88% of the CDSs can be unique to a fungus in comparison to another fungal species. Nearly 50% of the CDSs of *A. niger *CBS 513.88 cannot be found in other *Aspergillus *species (refer to Materials and methods for the definition of uniqueness). The CDSs are well conserved in the two *A. niger *strains. Over 30% of the CDSs in each *A. niger *strain have homologs in another *A. niger *strain with an identity level higher than 99%. The strain ATCC 9029 and the strain CBS 513.88 have 639 and 575 unique CDSs (Additional data file 3), respectively, in comparison with each other. The unique genes account for around 4% of the total number of CDSs in the two *A. niger *strains. Similar results were also achieved with the preliminary gene prediction of *A. niger *ATCC 1015 from the Joint Genomics Institute (the data are not shown because of the data release policy of the Joint Genomics Institute).

**Table 1:** Unique protein coding sequences (CDSs) in selected fungi revealed by comparative genomic analysis

			Unique CDSs in comparison to									
			
Strain	Abbreviation	Total CDSs	ands	anig	aor	afm	ani	fgra	dmgr	dncr	sce	All others
*A. niger *CBS 513.88*	ands	14,165		575	7,018	7,425	7,808	9,866	10,481	10,393	12,588	3,308
*A. niger *ATCC 9029	anig	13,937	639		6,857	7,147	7,499	9,685	10,285	10,216	12,387	3,039
*A. oryzae*	aor	12,059	4,858	5,013		5,320	5,714	7,644	8,306	8,335	10,511	3,392
*A. fumigatus*	afm	9,923	3,238	3,273	3,293		3,691	5,770	6,259	6,185	8,370	2,030
*A. nidulans*	ani	9,541	3,242	3,224	3,294	3,321		5,545	6,038	6,056	8,084	2,011
*F. graminearum*	fgra	11,640	7,381	7,458	7,288	7,451	7,613		6,654	6,748	10,137	4,863
*M. grisea*	dmgr	11,109	7,525	7,594	7,477	7,496	7,637	6,218		6,551	9,724	5,327
*N. crassa*	dncr	10,620	6,973	7,058	7,036	6,952	7,183	5,802	6,070		9,140	5,027
*S. cerevisiae*	sce	5,863	4,100	4,149	4,127	4,073	4,188	4,118	4,245	4,149		3,933

*As an example, 575 out of 14,165 CDSs in the strain *A. niger *CBS 513.88 do not have orthologs in the strain *A. niger *ATCC 9029; therefore, they are unique to *A. niger *CBS 513.88 in comparison to *A. niger *ATCC 9029.

The strain-specific genes in the two *A. niger *strains are listed in Additional data file 3. Among the genes unique to *A. niger *CBS 513.88, some encode enzymes for primary metabolism (such as alcohol dehydrogenase (NADP^+^) (An10g00010), fructose-1,6-bisphosphate aldolase (An16g00110), NADH dehydrogenase (An06g00130)), some for secondary metabolism (such as cephalosporin acylase, An16g00140), and some for transcription factors/regulators. A large gene cluster spans over 90 genes (from An08g11200 to An08g12140), of which 52 are unique to *A. niger *CBS 513.88 and most have unknown functions. Seven transposable elements are located in or next to this cluster, giving hints to its potential evolutionary origin by horizontal gene transfer. Interestingly, 25 of the CDSs unique to *A. niger *ATCC 9029, including glucokinase (Anig00906), UDP-N-acetylmuramoylalanine-D-glutamate ligase (Anig04708), UDP-N-acetylglucosamine-N-acetylmuramyl-(Pentapeptide) pyrophosphoryl-undecaprenol N-acetylglucosamine transferase (Anig04709) and five proteins involved in transport, have strong similarity (70-95% identical) to bacterial or bacteriophage proteins, indicating a possible bacterial origin of these proteins. The majority of the remaining genes unique to ATCC 9029 do not show any significant homology to the NCBI protein database.

In some cases the unique CDSs are just duplicates: their homologs can be found in both CBS 513.88 and ATCC 9029 (refer to Additional data file 3). For example, in addition to the unique gene An16g00110, CBS 513.88 has three further copies of genes coding for fructose-1,6-bisphosphate aldolase, An14g04410, An05g02040 and An02g07470, which are orthologous to the three copies of fructose-1,6-bisphosphate aldolase in ATCC 9029, Anig06338, Anig11911 and Anig08668, respectively.

In summary, the results from comparative genomics show that the *A. niger *strains are closely related to each other but exhibit large differences from the other fungal species compared. In the following paragraphs we address the impact of these differences on the metabolic peculiarities of *A. niger*.

### Reconstruction and comparative analysis of the metabolic network

#### Metabolic network reconstruction

**Figure 2:**
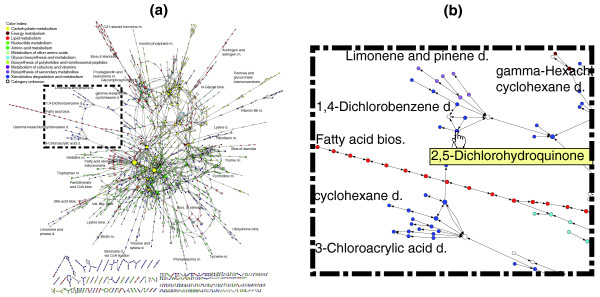
The genome-scale metabolic network of *A. niger*. Nodes are metabolites while links are reactions. The color of the nodes represents different functional categories. The size of nodes is proportional to the number of reactions from or to that node (metabolite) in the genome-wide network. **(a) **The general layout of the metabolic network. **(b) **A zoom-in of the dashed box in (a). For a detailed and clickable version, see Additional data files 5 and 6.

For the reconstruction of the metabolic network, only CDSs having standardized EC numbers were considered. From the functional annotation discussed above, 999 unique EC numbers (935 of them are complete) were identified in 4,006 CDSs. Similar EC numbers were also identified from the genome of *A. niger *CBS 513.88. The metabolic network of *A. niger *was constructed using the EC numbers of these two strains. Both the knowledge-based [23] and the connection-matrix-based methods [15] were applied, as stated in Materials and methods. Figure 2 shows the genome-wide metabolic network, in which nodes represent metabolites and links represent the reactions. A reaction map of the metabolic network, in which the nodes represent reactions and links the common metabolites of two successive reactions, is included in Additional data file 4. Their corresponding clickable versions in html format can be found in Additional data files 5 and 6.

The metabolic network established contains 2,443 biological reactions (31 of them are non-enzymatic reactions; refer to Additional data file 7 for a complete list of reactions and corresponding CDSs) and 2,349 metabolites, significantly higher than the number of reactions and metabolites known for this organism until now. Most of the reactions are connected to central metabolism, such as carbohydrate metabolism, amino acid metabolism, lipid metabolism, energy metabolism, and so on (Table 2). Interestingly, a significant number of reactions and metabolites belong to secondary metabolism or xenobiotics biodegradation, indicating the high metabolic potential of *A. niger *for production of secondary metabolites or for bioremediation, which is consistent with the experimental findings in the literature [3-6,24]. Moreover, around 20% of all the identified reactions or metabolites are still not associated with any metabolic category. Many of them belong to isolated parts of the overall metabolic network (Figure 2). The missing links could be either due to our limited knowledge on the reference metabolic reactions and enzymes or because of insufficient or wrong genomic annotation. Identification of the missing links should be an important focus in further functional genomic studies to enable us to fully exploit the metabolic capacity of *A. niger*.

**Table 2:** Distribution of reactions and metabolites of the inferred genome-wide metabolic network in different functional categories

Functional category	Reactions	Metabolites
Carbohydrate metabolism	311	290
Energy metabolism	106	90
Lipid metabolism	275	279
Nucleotide metabolism	146	109
Amino acid metabolism	412	421
Metabolism of other amino acids	78	122
Glycan biosynthesis and metabolism	80	71
Biosynthesis of polyketides and nonribosomal peptides	8	28
Metabolism of cofactors and vitamins	157	181
Biosynthesis of secondary metabolites	129	196
Xenobiotics biodegradation and metabolism	218	298
Category unknown	418	444

A reaction or a metabolite may be involved in different metabolic pathways and, therefore, could be counted more than once.

#### A comparative assessment of the central metabolic network

The metabolic network reconstructed from the genomic data was compared to the network of central carbon metabolism of *A. niger *reconstructed by David *et al*. [17]. The network of David *et al*. was mainly based on literature data of *A. niger *and the genomic information of *A. nidulans *and other fungi. It contains 335 reactions, 284 metabolites and 129 EC numbers. In general, there is a good agreement between these two metabolic networks regarding central metabolism. Only 14 ECs in the metabolic network of David *et al*. could not be found in the genome-wide network reconstructed by us in this work, most of which belong to enzymes poorly characterized in the literature in terms of protein sequences. The reason for such minor discrepancies is discussed in detail in Additional data file 8.

#### Unique enzyme-coding genes and unique EC numbers

**Figure 3:**
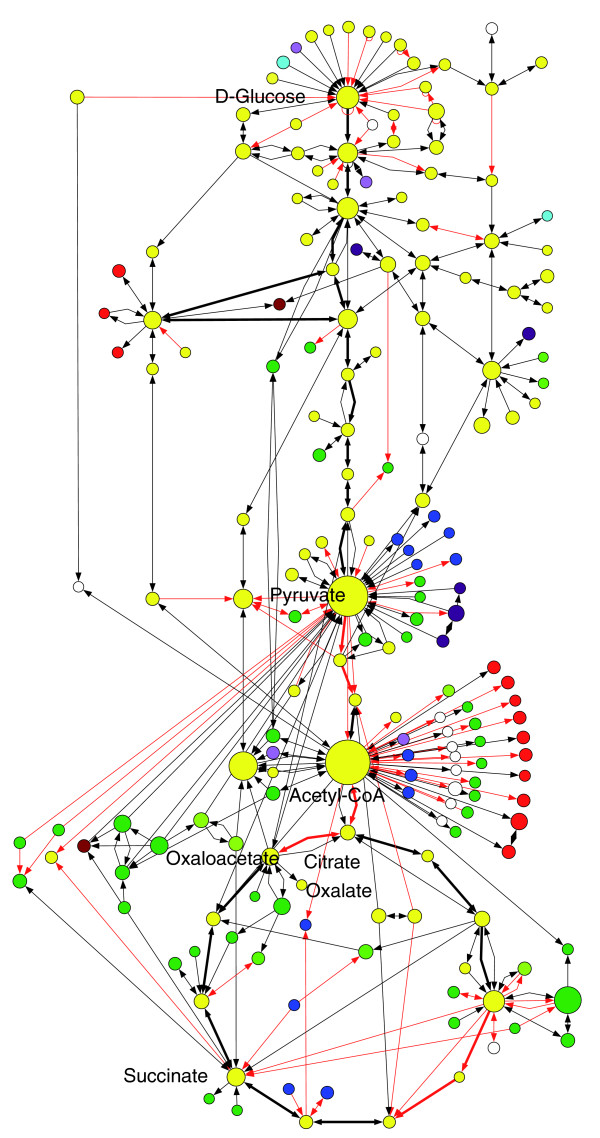
Glycolysis and TCA cycle of *A. niger*: a view from the genome-scale network. Nodes represent metabolites while directional links represent metabolic reactions. The color of the nodes represents different functional categories. The size of nodes is proportional to the number of reactions from or to that node (metabolite) in the genome-wide network. The red colored links indicate that *A. niger *has additional copies of genes for these reactions (see Additional data file 12 for details).

Based on the established ortholog relationship and in comparison to seven other fungi, 42 enzyme-coding ortholog groups are unique to one of the two *A. niger *strains (Additional data file 9), while 1,100 enzyme-coding orthologs were found to be common in the two *A. niger *strains and unique to them (Additional data file 10). Most of these common and unique genes have EC numbers that are also found in other fungi (for example, refer to Figure 3, red links). Additional or different copies of genes can strengthen certain pathways or enhance the robustness of the regulation to adapt to different environments [25,26]. Surprisingly, merely nine ortholog groups have EC numbers that were not found in the other fungi compared (Table 3), including two enzymes involved in secondary metabolism and three (EC 1.13.11.3, EC 4.1.1.55 and EC 1.3.1.11) associated with degradation of aromatic compounds. This is consistent with the fact that *A. niger *can be used for bioremediation to degrade aromatic compounds [27].

**Table 3:** Unique enzymes of *A. niger *in comparison to selected filamentous fungi

EC no.	CBS 513.88	ATCC 9029	KO no.	KO definition	Closest homolog in *A. niger *CBS 513.88	E-value	Identity (%)	Closest homolog in other Aspergilli	E-value	Identity (%)	Functional category
1.3.1.11	An12g02790	*		Coumarate reductase	An12g02420	5E-43	36	Afu5g09450	3E-57	39	Phenylalanine degradation
2.3.1.18	An13g03730	Anig05994		Galactoside O-acetyltransferase	An01g14790	9E-22	44	MG02103	1E-27	50	Carbon metabolism
3.6.3.41	An05g02470	Anig06282 Anig10968	K02021	ABC transport system ATP-binding protein	An08g04860	0	33	FG02316	0	30	Transport
5.4.99.-	An08g09210	Anig02930	K01865	S-adenosylmethionine tRNA ribosyltransferase							tRNA modification
4.2.1.94	An08g09920	Anig07347		Scytalone dehydratase				FG06477	7E-17	32	Biosynthesis of melanin
4.2.3.19	An11g06270	Anig08665	K04121	Ent-kaurene synthase	An18g02710	1.5E-67	31	AN1594	3E-78	31	Diterpenoid biosynthesis
2.5.1.39	An10g00130	Anig10859	K03179	4-Hydroxybenzoate octaprenyltransferase	An16g02750	7E-56	41	FG10613	7E-20	31	Ubiquinone biosynthesis
1.13.11.3	An02g11530	Anig09276	K00449	Protocatechuate 3,4-dioxygenase, beta subunit	An01g12310	2.7E-71	46	AN9363	3E-73	46	Benzoate and 2,4-dichlorobenzoate degradation
4.1.1.55	An15g07340	Anig08580	K04102	4,5-Dihydroxyphthalate decarboxylase							2,4-Dichlorobenzoate degradation

*: a genomic sequence nearly identical to An12g02790 was found in *A. ngier *ATCC 9029 but not annotated as a gene by the automatic genome annotation procedure described in Materials and methods.

It should be noted that in most cases, the unique enzyme-coding genes mentioned above do have paralogs in other fungi or even in *A. niger *itself (refer to Materials and methods for the definition of uniqueness). These paralogs were carefully verified not to be orthologs since they are orthologous to other CDSs of *A. niger*. Gene redundancy or duplication has also been reported in *A. niger *previously [13,28], and is commonly found in eukaryotes [26,29,30]. Due to slackened selective constraints, the duplicated genes have greater potential for mutation to undergo slight changes in function, such as different substrate or ligand specificity, to achieve different temporal or spatial distribution, to be differently regulated, or even to gain completely new functions [26]. It is interesting to ask what are the biological functions of these unique but paralogous enzymes in *A. niger*. As can be seen in Table 3, only two enzymes of *A. niger *have no homolog in the other fungi, namely 4,5-dihydroxyphthalate decarboxylase (EC 4.1.1.55), involved in 2,4-dichlorobenzoate degradation, and S-adenosylmethionine tRNA ribosyltransferase (EC 5.4.99.-), involved in tRNA modification. The finding concerning S-adenosylmethionine tRNA ribosyltransferase is somewhat surprising, because this enzyme is exclusively present in eubacteria for *de novo *biosynthesis of queuosine, which is an essential nutrient for many eucaryotes [31,32] (see Additional data file 11 for a detailed analysis).

### Citric acid production as a case study

#### A versatile metabolic conversion center

In view of the importance of citric acid production by *A. niger*, the metabolic reactions contributing to citric acid production are selected as an example to explore the capability of the constructed metabolic network. Although citric acid production has been studied extensively in the past, there are still many questions that need to be answered to fully understand the citric acid formation process [2]. The pathways related to citric acid production from glucose were extracted from the genome-wide metabolic network together with the metabolites directly connected to these pathways (Figure 3; refer to Additional data file 12 for details). The extensive connections of the 25 intermediates of glycolysis/tricarboxylic acid (TCA) cycle from/to the 146 metabolites of other pathways demonstrate the complexity and large interactions of the central metabolism. Eighteen substrates, such as starch, sucrose, dextrin, maltose, lactose, cellulose, α,α-trehalose, sorbitol, D-glucoside, N-glycan, and so on, require only a one-step reaction to enter this pathway via glucose. In comparison to other filamentous fungi, *A. niger *has redundant unique genes for the conversion of seven of these substrates (reactions marked as red in Figure 3). Degradation products from many pathways, including xenobiotic and amino acid metabolism, enter this citrate biosynthesis sub-network via pyruvate or acetyl-CoA for further processing. Acetyl-CoA and pyruvate belong to the metabolites having the highest connectivity (involved in 65 and 57 reactions, respectively) in the metabolic network of *A. niger*. They are directly used for biosynthesis of amino acids, lipids, vitamins, and so on. 2-Oxoglutarate and dihydroxyacetone phosphate from this sub-network are two other metabolites involved in many pathways for biosynthesis (lipids and amino acids, respectively). Anaplerotic pathways of the TCA cycle were identified from the metabolic map, including reactions from the glyoxylate cycle, from phosphoenolpyruvate to oxaloacetate, or from pyruvate to oxaloacetate or malate. The versatile conversion center TCA cycle can potentially offer a sufficient amount of oxaloacetate for the formation of oxalic acid, often an unwanted acidic by-product in *A. niger *cultivations.

#### Additional copies of genes encoding alternative mitochondrial oxidoreductase and citrate synthase in the citric acid production strain

**Figure 4:**
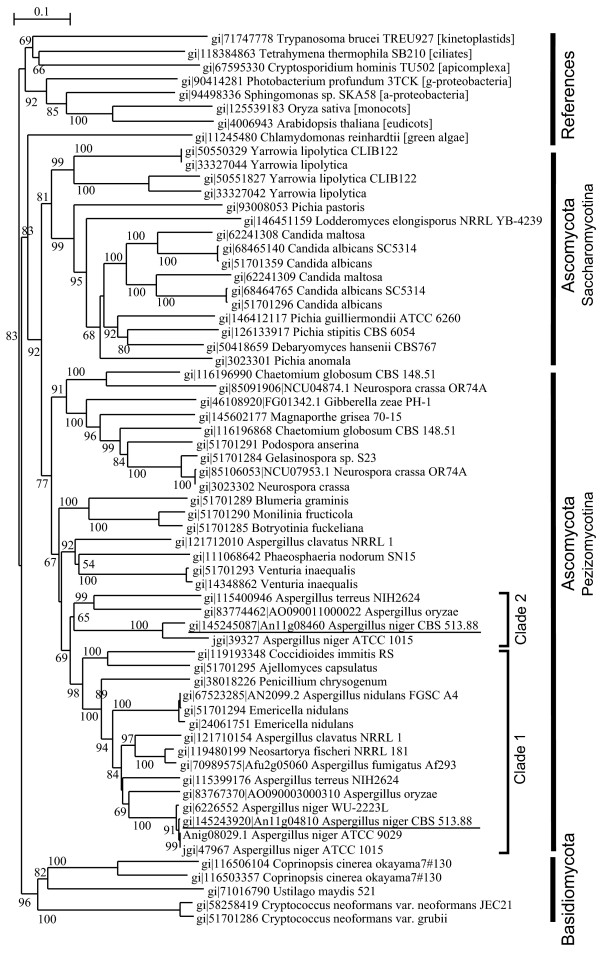
Phylogenetic analysis of fungal alternative oxidases (AOX). An11g08460 and An11g04810 were used as a query to search in the NCBI nr database for retrieving homologs (cutoff E-value 1E-10, partial sequences ignored). Fungal AOX homologs and the reference non-fungal AOX sequences were aligned to build a phylogenetic tree with 1,000 bootstraps (bootstrap value shown in percentages), using the software ClustalW [52].

Thirty percent of all the reactions (marked red in Figure 3) can be potentially catalyzed by enzymes encoded by additional or different CDSs that are unique to the *A. niger *strains in comparison to other filamentous fungi. Two examples are given here. The first one is the cyanide-insensitive and salicylhydroxamic acid-sensitive mitochondrial alternative oxidoreductase (AOX, EC 1.9.3.-,), which may have a critical role in the citric acid production process due to the necessity to rapidly recycle NADH independent of the electron transport chain and ATP synthesis [2,33-35]. Inhibition of AOX by adding salicylhydroxamic acid into the media greatly reduces citric acid production [34,35]. Previously, only a copy of AOX (gi|6226552|AOX_ASPNG from SwissProt, 99% identical to An11g04810) was experimentally identified in *A. niger *by using cDNA cloning and genomic Southern blot hybridization [33,34]. Interestingly, we have now identified an additional mitochondrial AOX, 67% identical to the first one, from the genomes of the three *A. niger *strains (Table 4, ortholog index number 10903), which is unique to *A. niger *in terms of orthology. Phylogenetic analysis of AOX homologs revealed that these two copies fall into two neighbored clades (clades 1 and 2 in Figure 4) belonging to Pezizomycotina. Clade 1 includes the known copy of AOX in *A. niger *and the AOX from *Penicillium chrysogenum*, *Ajellomyces capsulatus*, *Coccidioides immitis*, *Neosartorya fischeri *and all sequenced Aspergilli, while clade 2 includes the second copy of AOX found in *A. niger*, *A. oryzae *and *A. terreus*. Multiple copies of AOX were also found in some fungi, such as *Neurospora crassa*, *Chaetomium globosum*, *Candida maltosa*, *Candida albicans*, and *Yarrowia lipolytica *(Figure 4), and in many plants [36]. The different copies of AOX in plants are expressed in different environmental or developmental conditions [36]. In *A. niger*, the AOX (gi|6226552|AOX_ASPNG) identified previously seems to be constitutively expressed regardless of the glucose concentration at 10-120 g/l in the cultivation media [37]. The participation of the newly identified AOX as an important enzyme in the citric acid formation process would need further experimental verification.

**Table 4:** Distribution of alternative mitochondrial oxidoreductase and citrate synthase genes in *Aspergillus *and selected fungi

Ortholog group	CBS 513.88	ATCC 9029	ATCC 1015*	aor	afm	ani	fgra	dmgr	dncr
**AOX**									
3125	An11g04810	Anig08029.1	47967	AO090003000310	Afu2g05060	AN2099.2	FG01342.1		NCU07953.2
10903	An11g08460	Anig03716.1	39327	AO090011000022					
									
**CS**									
361	An09g06680	Anig07591.1	202801	AO090102000627	Afu5g04230	AN8275.2	FG01422.1	MG07202.4	NCU01692.2
2397	An15g01920	Anig05911.1	48684	AO090009000568	Afu6g03590	AN6650.2	FG00175.1	MG02617.4	NCU02482.2
6051	An09g03570	Anig12406.1	126525	AO090012000318	Afu2g15310	AN7593.2			
7402	An08g10920	Anig08443.1	176409	AO090010000170			FG02352.1		
12065	An01g09940	Anig10631.1	35756						
			46236						

*The complete identifier for the genes of *A. niger *ATCC 1015 is 'jgi|Aspni1|' plus the number in this column. aor, *A. oryzae*; afm, *A. fumigatus*; ani, *A. nidulans*; fgra, *F. graminearum*; dmgr, *M. grisea*; dncr, *N. crassa*.

The second example is the citrate synthase (Table 4) catalyzing the biosynthesis of citric acid from acetyl-CoA and oxaloacetate. Table 4 shows the relevant orthologs across the selected fungi. The *A. niger *strains share five isoenzymes of citrate synthase, including methylcitrate synthase, which also shows citrate synthase activity [38], whereas *A. oryzae *has only four, and *A. fumigatus *and *A. nidulans *have only three copies. The genetic multiplicity of citrate synthase was also reported in *A. niger *[13]. Here, we identified that the ortholog group 12065 is unique to all three *A. niger *strains. Interestingly, *A. niger *ATCC 1015, the strain used in the first patented citric acid process, has an additional unique citrate synthase gene, jgi|Aspni1|46236 (Table 4). Its ortholog was not found in *A. niger *CBS 513.88 or *A. niger *ATCC 9029. The sequence of this protein is identical to gb|EAV74068.1, citrate synthase I of *Delftia acidovorans *SPH-1 (β-proteobacteria). This partial gene is found on a small contig, scaffold_1409, whose nucleotide sequence is also identical to the genomic sequence of *Delftia acidovorans *SPH-1. The presence of this additional and bacteria-originated citrate synthase in *A. niger *ATCC 1015 needs to be carefully verified by genome sequencing or PCR to rule out the possibility of sequence contamination. Furthermore, a detailed phylogenetic analysis of 1,134 homolog sequences (homologous to any of the six groups of citrate synthase in *A. niger *at E-value 1E-20) from the NCBI nr protein database showed that the sequences of the first two ortholog groups (361 and 2397) in Table 4 are clearly clustered with the sequences mainly from eukaryotes while those of the last four ortholog groups are clustered with prokaryotic sequences (Additional data file 13), suggesting different origins of citrate synthase in *A. niger*. Since the members of the *A. niger *unique ortholog group 12065 is tightly clustered with the other two ortholog groups, these genes probably originated after speciation by gene duplication events. As revealed by the analysis above, gene duplication or redundancy seems to be a general strategy evolved in this black mould. These newly found extra copies of genes in *A. niger *strains, most remarkably the second AOX and the additional citrate synthases, may contribute to the high citrate production efficiency of *A. niger*.

## Conclusion

14,000 protein coding sequences were predicted from the raw low-coverage genome sequence of *A. niger *ATCC 9029 and approximately 60% of them were assigned to at least one functional category (GO, KO, COG, EC and pathways). This enabled a comparative genomic analysis of two different *A. niger *strains. It is found that the genomic content of *A. niger *ATCC 9029 is very similar to that of *A. niger *CBS 513.88; merely around 600 genes are exclusively found in each strain. A further comparative genomic analysis among six Aspergilli and other selected eucaryotes revealed more than 4,000 CDSs unique to *A. niger*. Based on the functional annotation of the two *A. niger *strains, we reconstructed the metabolic network of *A. niger *and systematically compared it with those of seven other fungi. Comparative metabolic genomics revealed the high metabolic peculiarity of *A. niger *by more than 1,100 unique enzyme-encoding genes. Many of these unique genes are additional copies (paralogs) of those genes that are common (orthologs) in the compared fungi, indicating that genetic multiplicity might be a key strategy of *A. niger *to keep its versatile metabolic capacities and its robustness to adapt to different environmental conditions. Only nine genes were identified to encode enzymes with EC numbers exclusively found in *A. niger*, mostly involved in the biosynthesis of complex secondary metabolites and degradation of aromatic compounds. Moreover, we identified additional copies of genes, such as the ones encoding alternative mitochondrial oxidoreductase and citrate synthases, which could have an impact on the overproduction of citric acid by this black mould.

## Materials and methods

### Genome sequence of *A. niger*

The genome (approximately 32 Mb, haploid 8 chromosomes) sequence of *A. niger *ATCC 9029 was obtained from Integrated Genomics Inc (Chicago, IL, USA), which has a genome coverage of about three-fold and was generated by using a whole-genome shotgun strategy without finishing. The assembled genomic sequence contains 9,510 contigs corresponding to 33.7 Mb. The average length of the contigs is 3.5 kb. The largest contig is 34.9 kb. The genome of *A. niger *CBS 513.88 and its annotation were kindly provided by the company DSM ahead of publication [13]. The genome of *A. niger *ATCC 1015 and its gene prediction were downloaded from the Joint Genome Institute under its data release agreement [18]. Genome sequences of other fungal strains were downloaded from KEGG and NCBI.

### Prediction and annotation of protein-coding sequences

To predict the CDSs and to reconstruct the metabolic network of organisms with unannotated, low coverage genome sequences, we recently developed a new algorithm called 'IdentiCS' [23]. This homology-based algorithm was demonstrated to be able to cope with sequences of low genome coverage and, thus, potentially high error rates. It was successfully used to predict CDSs and to infer the metabolic networks of several bacteria, including *Klebsiella pneumoniae *and *Salmonella typhimurium *[23], *Escherichia coli *1917 [39] and *Bacillus megaterium *[21]. In this study, this algorithm was extended for the prediction and annotation of eukaryotic CDSs by considering the intron and extron structure of genes (see Additional data file 1).

### Complementation of 'IdentiCS' by GenScan and GeneWise for the prediction of protein-encoding genes

GeneWise, another useful software applying a homology-based approach to predict gene structure [19], was used to refine and confirm the prediction of 'IdentiCS' as described above. Like all other homology-based methods, 'IdentiCS' is unable to predict new genes for which no homologue is present in the available protein database. Thus, a homology-independent program called GenScan was used. GenScan is a general-purpose gene identification program that determines the most likely 'parse' (gene structure) by using a probabilistic model of the gene structural and compositional properties of the genomic DNA for the given organism [20].

### Refinement of the annotation

We combined several strategies to refine the annotation. First, all CDSs predicted were submitted to the KEGG Automatic Annotation Server [40] which applies a best-best algorithm to associate the submitted sequence to known KO number, COG, EC number, GO number and biochemical reactions. Second, we used HT-GO-FAT (High Throughput Gene Ontology Functional Annotation Toolkit) [41], another useful software toolkit that utilizes a custom-curated BLAST database to annotate sequences to GO, EC number, KEGG pathways and so on. EC numbers can be deduced from the associated GO numbers by this program. Third, text mining was used to assign EC number when an obvious enzyme could not be associated to an EC number or a complete EC number through the above-mentioned methods. For this purpose, the name of the enzyme was queried in the KEGG Ligand database [42,43] for synonyms or searched via a general search engine such as Google [44]. The hits were manually evaluated.

### Comparative genomics

Proteins predicted from the unfinished genomic sequences of *A. niger *ATCC 9029 and the proteins from *A. niger *CBS 513.88 were cross-compared with the proteins of seven selected fungal species and another 26 representative eukaryotic organisms to identify their orthologous relationships. The seven selected fungi included *A. oryzae *(used in Asian food fermentation), *A. fumigatus *(a human pathogen), *A. nidulans *(a model organism for genetic studies), *F. graminearum *(a plant pathogen but also used in food production), *Magnaporthe grisea *(a plant pathogen), *N. crassa *(a model organism) and *S. cerevisiae *(used in baking and brewing but also a model organism). The protein ortholog relationship among *A. niger *and the selected fungi was detected by the program OrthoMCL [22] with relatively strict parameters, such as *p *value cutoff 1E-20, identity cutoff 40%, percentage-of-match cutoff 50% and inflation factor 5. OrthoMCL detects the many-to-many ortholog groups including recent paralogs based on all-against-all sequence alignment. This algorithm is suitable to work with more genomes. Blast [45] and PatternHunter [46] were used for sequence alignment.

Comparative genomics revealed a huge number of species-specific genes, even when two closely related sub-species were compared to each other. We found 3,976 CDSs unique to *A. niger *ATCC 9029 compared to *A. niger *CBS 513.88, and 4,306 unique to *A. niger *CBS 513.88 compared *vice versa*. We argue that this number is too high to be true because the sequence alignment of the two subspecies usually showed very high identity (close to 100%), indicating that the speciation is really a recent evolutionary event and a huge difference in genomic content is thus impossible. Failure of gene prediction in the genome annotation process can cause failure in ortholog detection. To avoid this problem, the protein sequence of each *A. niger *strain was compared against the genomic sequences of the other *A. niger *strain by applying strict criteria (aligned region >80% and identity >80% or log10(e2) < 0.8*log10(e1), where e2 is the E-value of the query protein against the genome while e1 is the E-value of the query protein against itself) in addition to the criteria mentioned above. This procedure ensures the detection of near-identical genes (ortholog or inparalog [22]) even in cases where they were not predicted as genes in one of the compared strains by the initial genome annotation process. The results were surprising: many nearly identical genomic regions were predicted as genes in *A. niger *ATCC 9029 but not in *A. niger *CBS 513.88, or *vice versa*, strongly demonstrating the necessity for improvement of current gene finding strategies, for instance, by integrating results from comparative genomics study. By this procedure, the number of genes truly specific to one of the *A. niger *strains is greatly reduced (see Table 1 for the results). This procedure was not applied for the cross-comparison of *A. niger *with the other fungi due to lack of an operational criterion.

### Uniqueness of genes or CDSs

In this work, if not specified, the uniqueness of a gene or CDS from organism A is defined according to the orthologous relationship table established above. If the ortholog of a gene from organism A is absent in organism B, we define that this gene is unique or specific to organism A in comparison to organism B. This does not imply there is no homolog (namely paralog) of the gene from organism A in organism B. In some cases, this gene is just an additional copy of another gene whose orthologs are found in both organisms. This also does not imply that this gene is found only in organism A. For example, the ortholog of this gene may be found in organism C from the relationship table or another strain or species that is not compared in this work.

### Metabolic network reconstruction

Two methods were used to reconstruct and visualize the metabolic network based on the annotation of CDSs. One method was based on mapping the annotation information to knowledge bases such as the KEGG pathways through 'IdentiCS' [23]. The metabolic pathways and network reconstructed this way are intuitive with respect to our knowledge on biochemical pathways. They can be used for a straightforward comparison with the network of other sequenced organisms [23]. The metabolic network was also constructed based on the connection matrix of reactions according to Ma and Zeng [15]. Thirty-one non-enzymatic reactions were also considered in the latter approach. The connection matrix of reactions published by Ma and Zeng [15] was substantially improved in this work by: updating the enzyme reaction database to the newer version of KEGG Ligand (Status Nov. 2005) [42]; integrating the information of reversibility from KEGG pathway maps (Status Nov. 2005) [47] and confirming it with the BRENDA enzymatic database [48]; and considering the complete set of reactions available in the Ligand database. In the end, the new version contains 6,442 reactions instead of the 3,805 in the former version. In addition, half of the reactions from the former version were updated in term of reversibility and connection pairs (Additional data file 14; also, check the authors' website [49] for an updated version). The software Cytoscape [16,50] and yEd (a Java Graph Editor from the company yWorks) [51] were used as layout tools for the genome-wide network. The phylogenetic tree was built by using the software ClustalW (version 1.83 for Windows) [52].

## Abbreviations

AOX, alternative oxidoreductase; CDS, coding sequence; COG, Clusters of Orthologous Groups; EC number, Enzyme Commission number; GO, Gene Ontology; KO, KEGG orthology; TCA cycle, tricarboxylic acid cycle.

## Authors' contributions

JS carried out genomic annotation, comparative genomics analysis, metabolic network reconstruction and analysis, and drafted and finalized the manuscript. XL participated in the analysis of unique enzymes. UR and AZ initiated and supervised this study. All authors have contributed to writing the manuscript and approved it.

## Additional data files

The following additional data are available with the online version of this paper. Additional data file 1 describes the methods for prediction and annotation of protein-coding sequences. Additional data file 2 is a table listing the orthologous groups of genes from selected fungi and their functions. Additional data file 3 is a table listing the strain-specific CDSs identified by cross-comparing the two *A. niger *strains. Additional data file 4 shows the genome-wide metabolic network of *A. niger *as a reaction graph where nodes are reactions and links are common reactants (substrate or product) of two successive reactions. The color of the nodes represents different functional categories. Additional data file 5 is a clickable version of Figure 2 where nodes (metabolites) are linked to the KEGG Ligand database for detailed information. Additional data file 6 is a clickable version of Additional data file 4 where nodes (reactions) are linked to the KEGG Ligand database for detailed information. Additional data file 7 is a table listing all the reactions used for reconstruction of the *A. niger *metabolic network. The corresponding EC numbers and genes are also shown. Additional data file 8 shows the results of a comparative assessment of the central metabolic network. Additional data file 9 is a table listing the enzyme-encoding genes unique to one of the two *A. niger *strains in comparison with the other selected fungi. Additional data file 10 is a table listing the enzyme-encoding genes common in both *A. niger *strains and unique to them in comparison with the other selected fungi. Additional data file 11 is a phylogenetic analysis of the S-adenosylmethionine tRNA ribosyltransferase. Additional data file 12 is a detailed version of Figure 3, showing a network view from glucose to citrate, including the names of all metabolites. Additional data file 13 is a phylogenetic analysis of citric acid synthases (CS), where 1,123 sequences from the NCBI nr protein database homologous to any of the six CSs of *A. niger *(cutoff E-value 1E-20, partial sequence ignored) were aligned together with the CSs from *A. niger *ATCC 9029 and ATCC 1015 to build the phylogenetic tree with 1,000 time bootstraps, using the software ClustalW. Part A is an overview while part B is the full phylogenetic tree with GI number, strain name, taxonomy, and bootstrap values. Additional data file 14 is the reaction database used for reconstruction of the metabolic network. Additional data file 15 is the protein database of *A. niger *ATCC 9029.

## Supplementary Material

Additional data file 1 Methods for prediction and annotation of protein-coding sequences. Click here for file

Additional data file 2 Orthologous groups of genes from selected fungi and their functions. Click here for file

Additional data file 3 Strain-specific CDSs identified by cross-comparing the two *A. niger *strains. Click here for file

Additional data file 4 Genome-wide metabolic network of *A. niger *as a reaction graph where nodes are reactions and links are common reactants (substrate or product) of two successive reactions. The color of the nodes represents different functional categories. Click here for file

Additional data file 5 A clickable version of Figure 2 where nodes (metabolites) are linked to the KEGG Ligand database for detailed information. Click here for file

Additional data file 6 A clickable version of Additional data file 4 where nodes (reactions) are linked to the KEGG Ligand database for detailed information. Click here for file

Additional data file 7 All the reactions used for reconstruction of the *A. niger *metabolic network. The corresponding EC numbers and genes are also shown. Click here for file

Additional data file 8 Results of a comparative assessment of the central metabolic network. Click here for file

Additional data file 9 Enzyme-encoding genes unique to one of the two *A. niger *strains in comparison with the other selected fungi. Click here for file

Additional data file 10 Enzyme-encoding genes common in both *A. niger *strains and unique to them in comparison with the other selected fungi. Click here for file

Additional data file 11 Phylogenetic analysis of the S-adenosylmethionine tRNA ribosyltransferase. Click here for file

Additional data file 12 A detailed version of Figure 3, showing a network view from glucose to citrate, including the names of all metabolites. Click here for file

Additional data file 13 Phylogenetic analysis of citric acid synthases (CS), where 1,123 sequences from the NCBI nr protein database homologous to any of the six CSs of *A. niger *(cutoff E-value 1E-20, partial sequence ignored) were aligned together with the CSs from *A. niger *ATCC 9029 and ATCC 1015 to build the phylogenetic tree with 1,000 time bootstraps, using the software ClustalW. Part A is an overview while part B is the full phylogenetic tree with GI number, strain name, taxonomy, and bootstrap values. Click here for file

Additional data file 14 The reaction database used for reconstruction of the metabolic network. Click here for file

Additional data file 15 The protein database of *A. niger *ATCC 9029. Click here for file
