# Supplementary material for: Metabolic peculiarities of Aspergillus niger disclosed by comparative metabolic genomics
Source: Genome Biol. 2007 Sep 4;8(9):R182. doi: 10.1186/gb-2007-8-9-r182 (PMC2375020; doi:10.1186/gb-2007-8-9-r182)
Supplement: Additional data file 1 — Methods for prediction and annotation of protein-coding sequences. [file gb-2007-8-9-r182-S1.doc]

**Prediction and annotation of protein-coding sequences**

To predict the protein-coding sequences (CDSs) and to reconstruct the metabolic network of organisms with unannotated, low coverage genome sequences we recently developed a new algorithm called “IdentiCS” [23]. This homology-based algorithm was demonstrated to be able to cope with sequences of low genome coverage and thus potentially high error rates. It was successfully used to predict CDSs and to infer the metabolic networks of several bacteria including *Klebsiella pneumoniae* and *Salmonella typhimurium* [23], *E. coli* 1917 [39] and *Bacillus megaterium* [21]. In this study, this algorithm was extended for the prediction and annotation of eukaryotic CDSs by considering the intron and extron structure of genes.

The prediction of protein coding sequences and the consideration of intron and extron structure of a gene were achieved by using a homology-based strategy as shown in Fig. SW1-1. A known protein from public database such as UniProt or the non-redundant protein database from NCBI (NCBInr), called Protein X in this example, was queried to the genomic sequence (DNA) database of *A. niger* using the “tblastn” program from the standalone sequence alignment tool BLAST, or other comparable tools such as “PatternHunter” from Bioinformatics Solution Inc. (Waterloo, Canada). In the example shown in Fig. SW1-1 the continuous non-overlapped sections of protein X, namely x1, x2, x3 and x4, are found to be homologous to the translated amino acid sequences of different sections of an error-containing genomic contig, e1, e2, e3 and e4, respectively. e1 is next to e2 in the contig. However, e1 and e2 have to be translated from different reading frames in order to achieve a high similarity to the corresponding sections of the protein X. This fact indicates a potential sequencing error (e.g. base pair deletion or insertion between section e1 and e2). Therefore, e1 and e2 most probably belong to a single extron. On the other side, e2, e3 and e4 are also non-overlapping but separated from each other by additional DNA sequences i1 and i2. This indicates that the sequence sections i1 and i2 are potential introns whereas e1, e2, e3 and e4 are extrons. Accordingly, a protein coding gene X composed of two introns and three extrons is identified. The coding sequence of the gene X can be deduced by linking all the identified extrons (e1-e2-e3-e4). This gene is thus initially annotated by transferring the information from the annotation of protein X and further examined with other algorithms as described below.

Contig

**Extron**

**Extron**

**Extron**

**Intron**

**Intron**

Gene X

Database protein X

Translation of 6 frames

**+1**

**+1**

**+3**

**+2**

**x2**

**x1**

**x3**

**x4**

**e2**

**e1**

**e3**

**e4**

**i2**

**i1**

Fig. SW1-1. A scheme showing the homology-based approach to predict a protein-coding gene and its intron-extron structure from raw erroneous genomic sequence.
