# Supplementary figures and images for: Metabolic peculiarities of Aspergillus niger disclosed by comparative metabolic genomics
Source: Genome Biol. 2007 Sep 4;8(9):R182. doi: 10.1186/gb-2007-8-9-r182 (PMC2375020; doi:10.1186/gb-2007-8-9-r182)

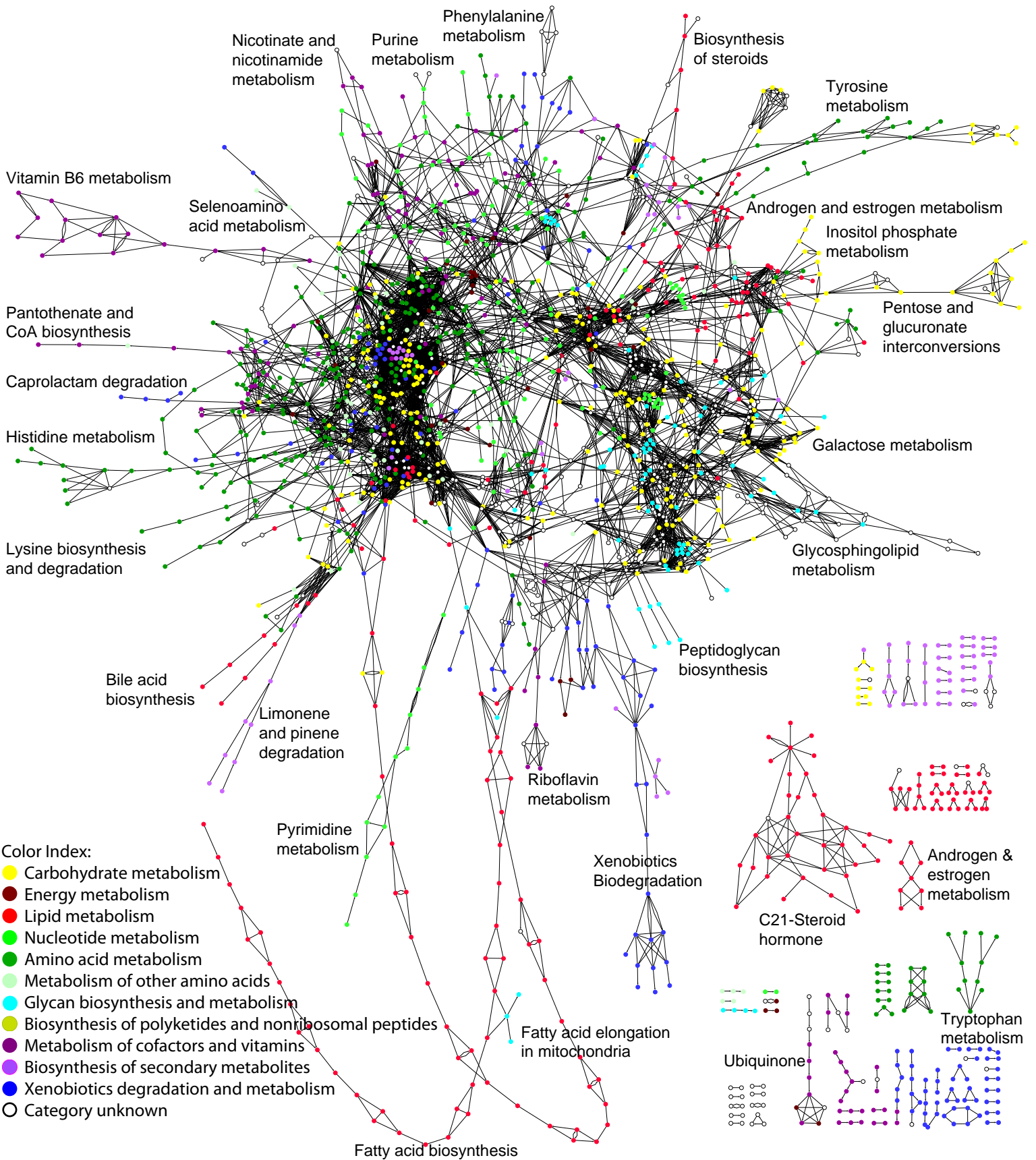

Supplement: Additional data file 4 — Genome-wide metabolic network of A. niger as a reaction graph where nodes are reactions and links are common reactants (substrate or product) of two successive reactions. The color of the nodes represents different functional categories. [file gb-2007-8-9-r182-S4.pdf]

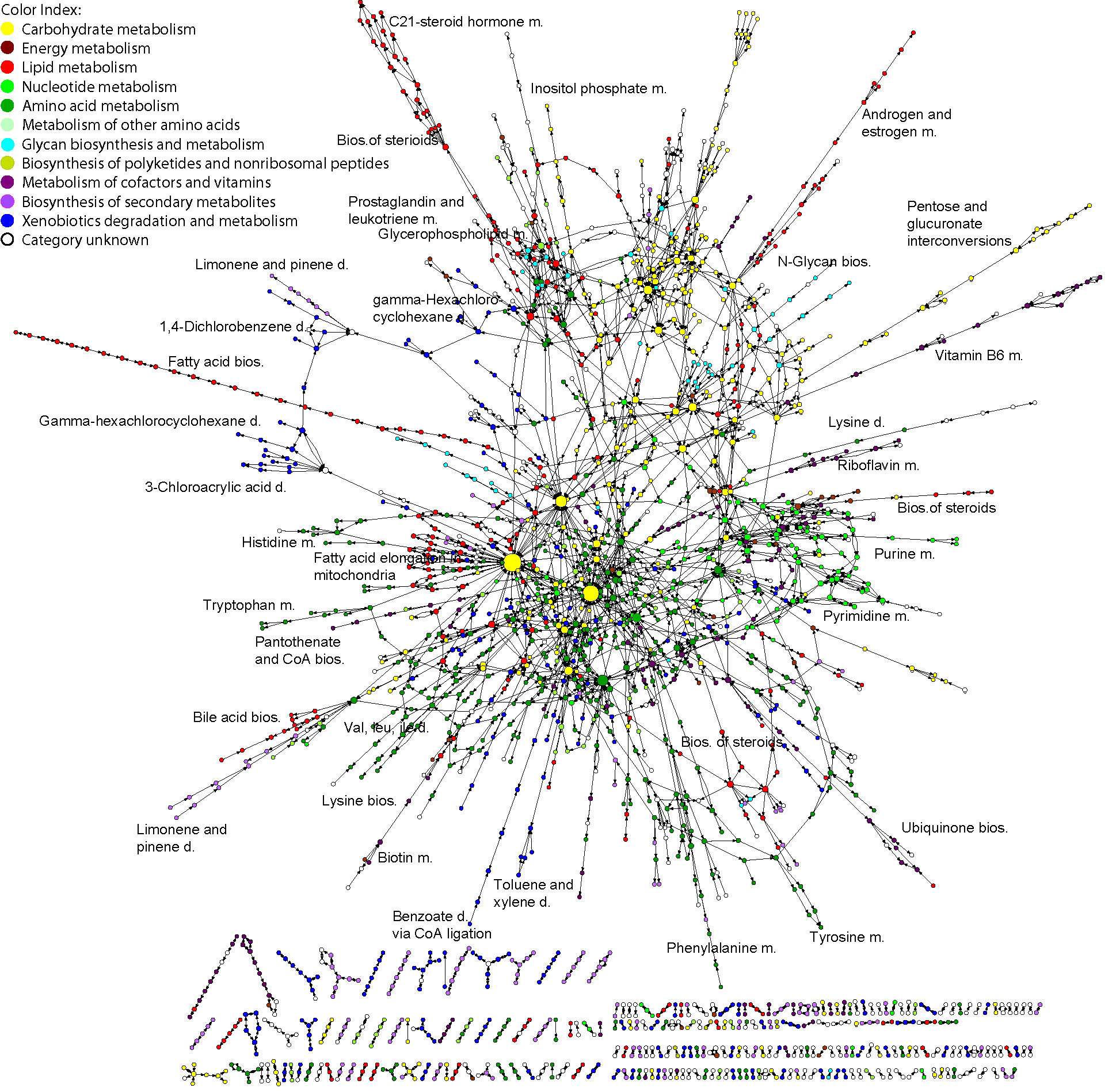

Supplement: Additional data file 5 — A clickable version of Figure 2 where nodes (metabolites) are linked to the KEGG Ligand database for detailed information. [file gb-2007-8-9-r182-S5.zip › Additional data file 5/A.niger.metgraph.jpg]

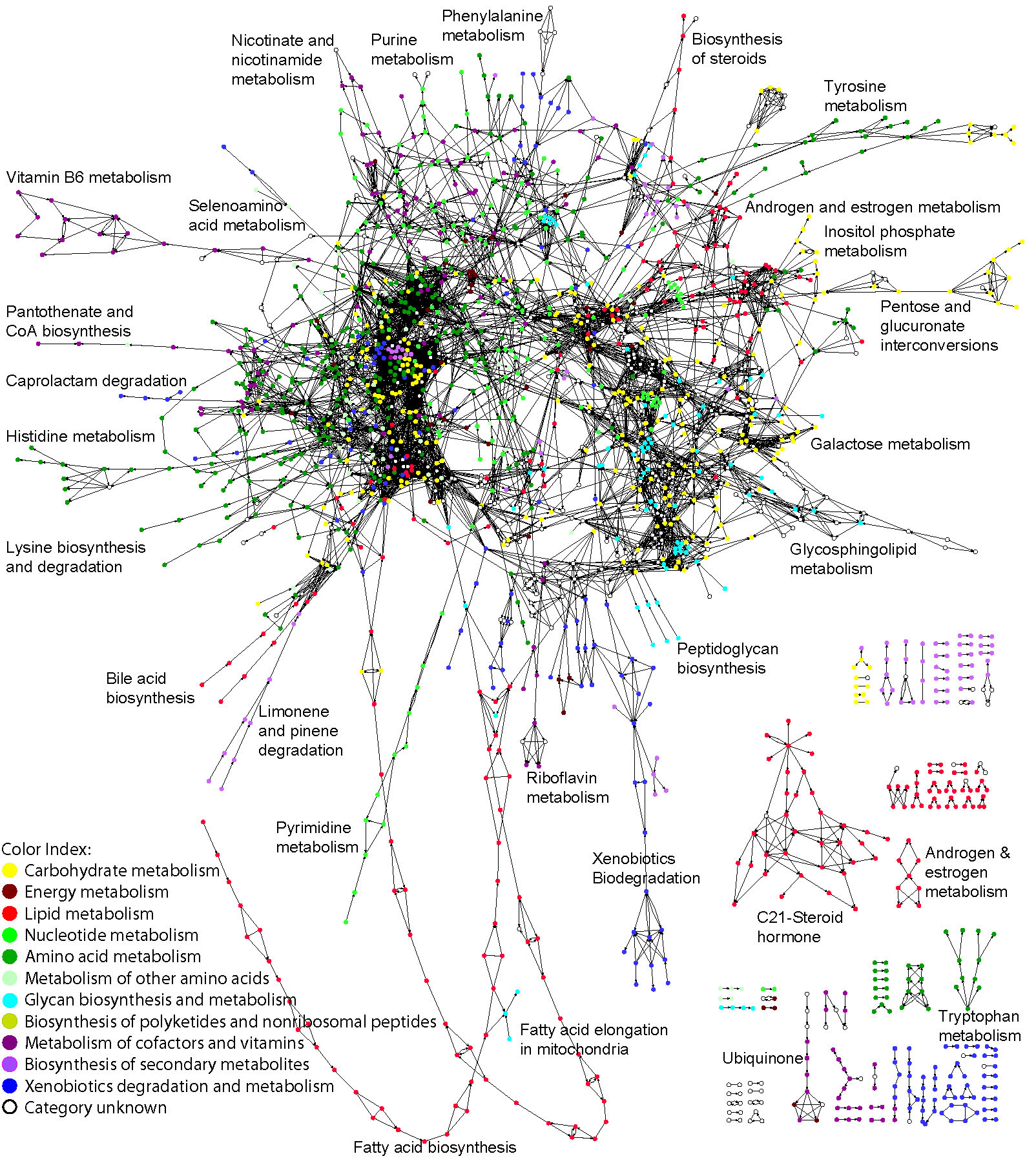

Supplement: Additional data file 6 — A clickable version of Additional data file 4 where nodes (reactions) are linked to the KEGG Ligand database for detailed information. [file gb-2007-8-9-r182-S6.zip › Additional data file 6/A_niger_MetNet_Reaction.jpg]

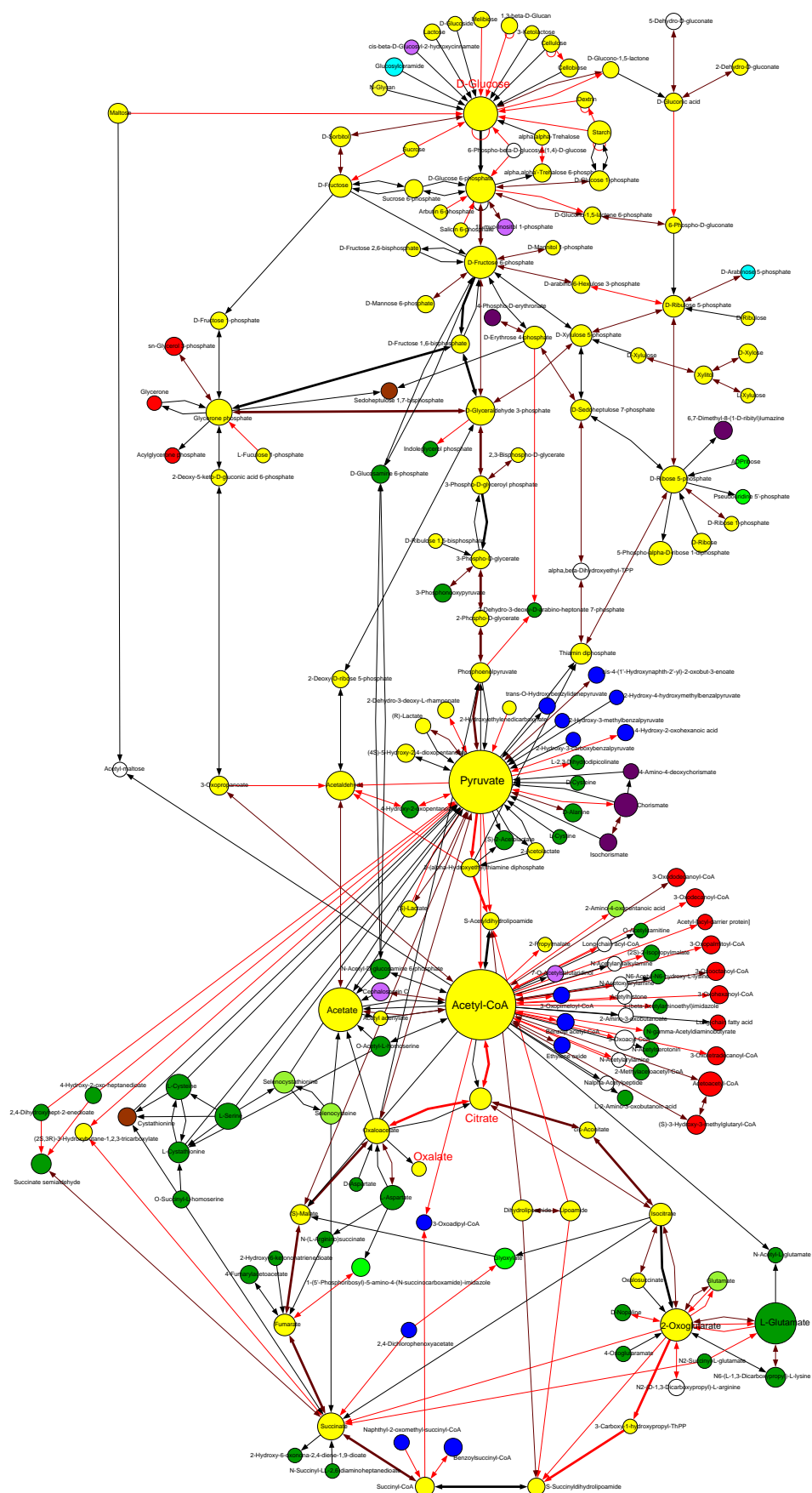

Supplement: Additional data file 12 — A detailed version of Figure 3, showing a network view from glucose to citrate, including the names of all metabolites. [file gb-2007-8-9-r182-S12.pdf]

Part A

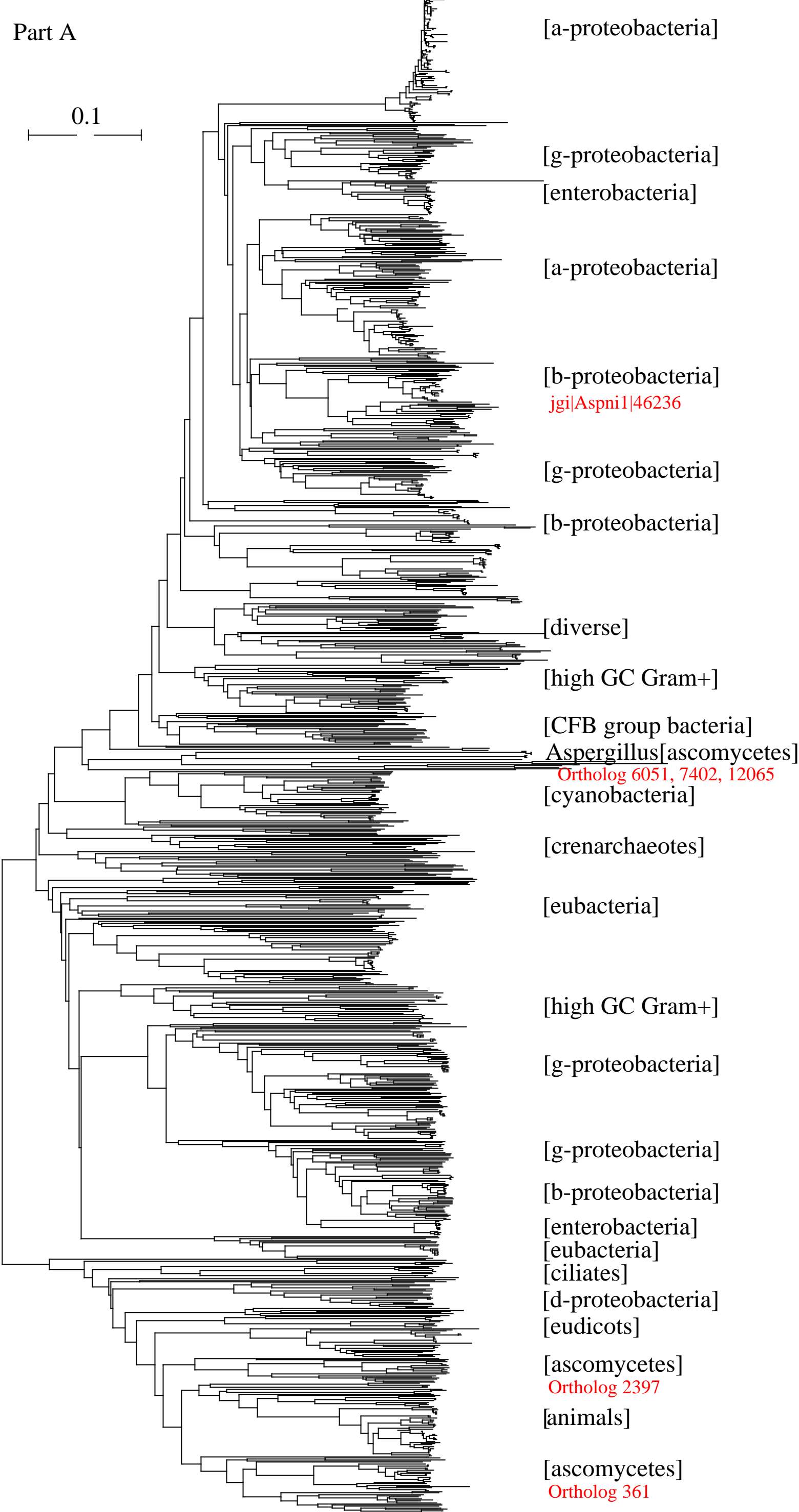

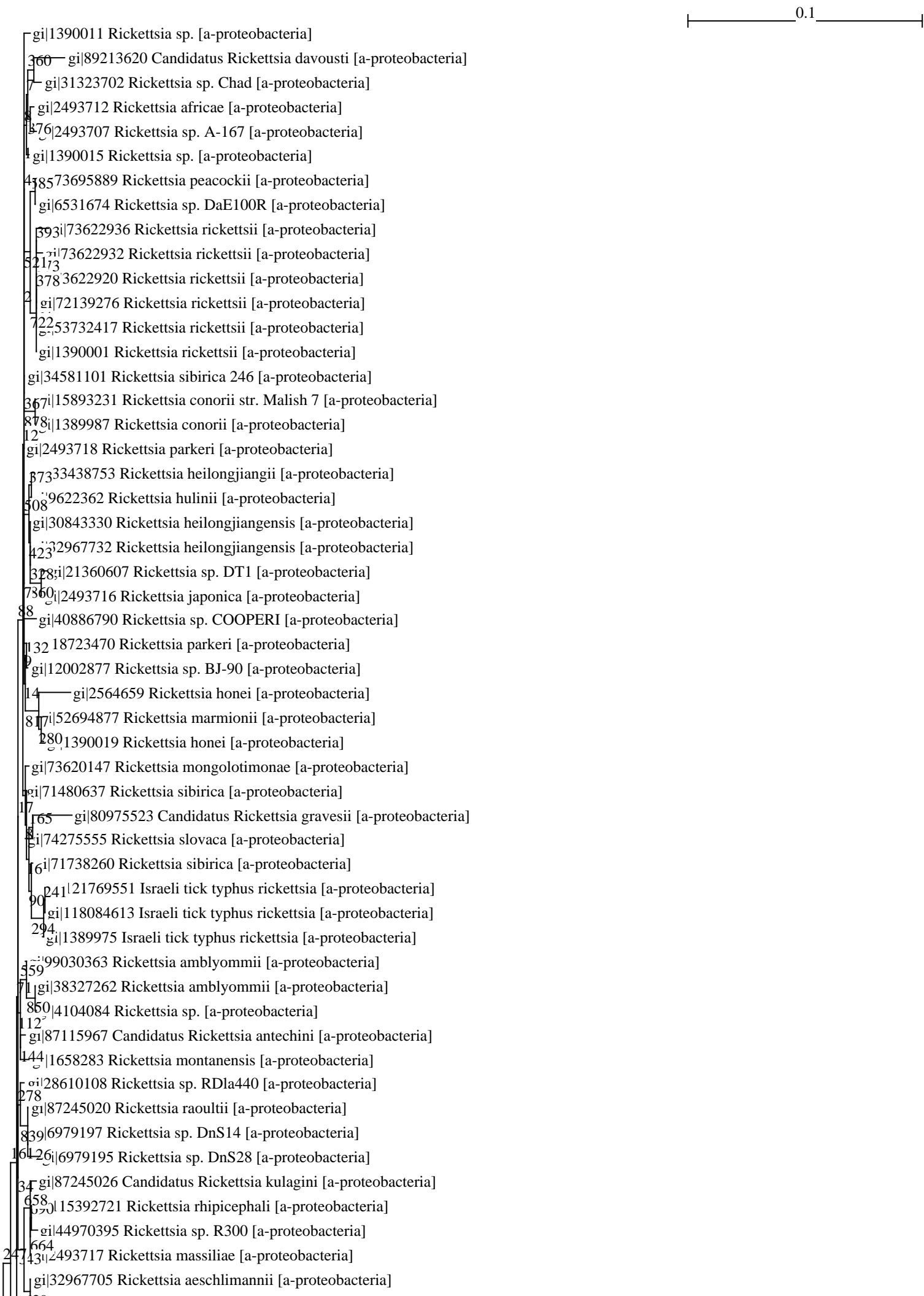

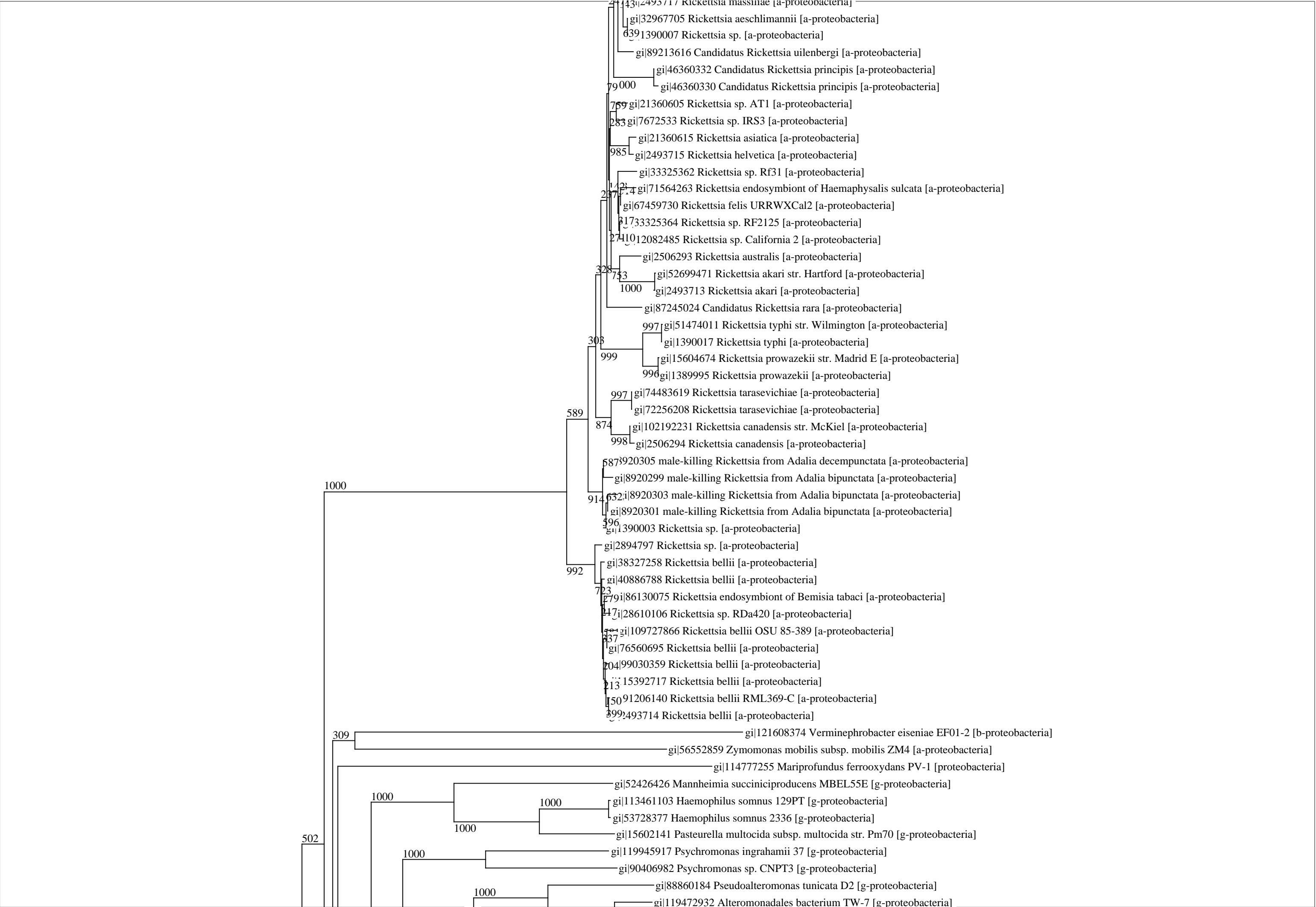

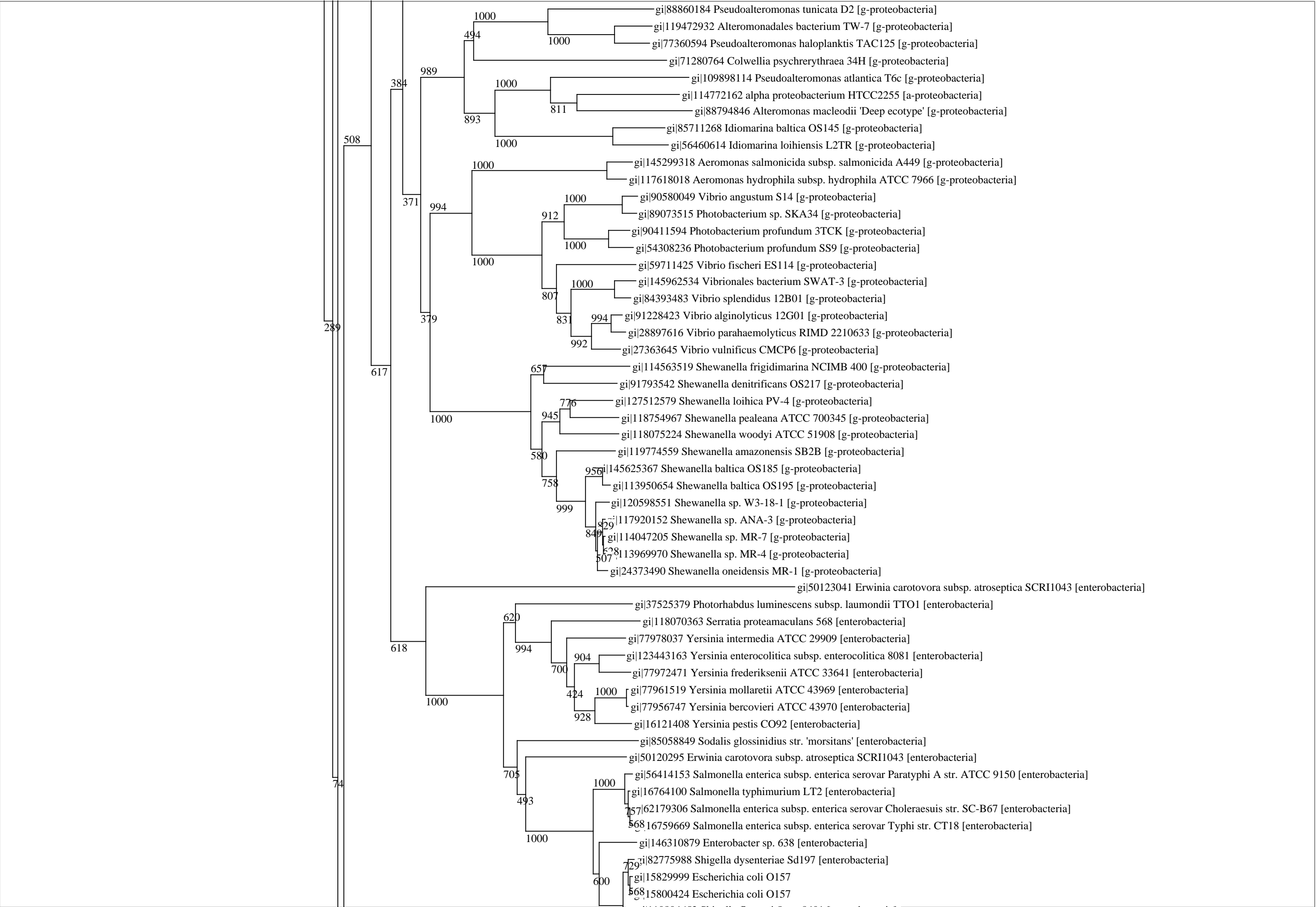

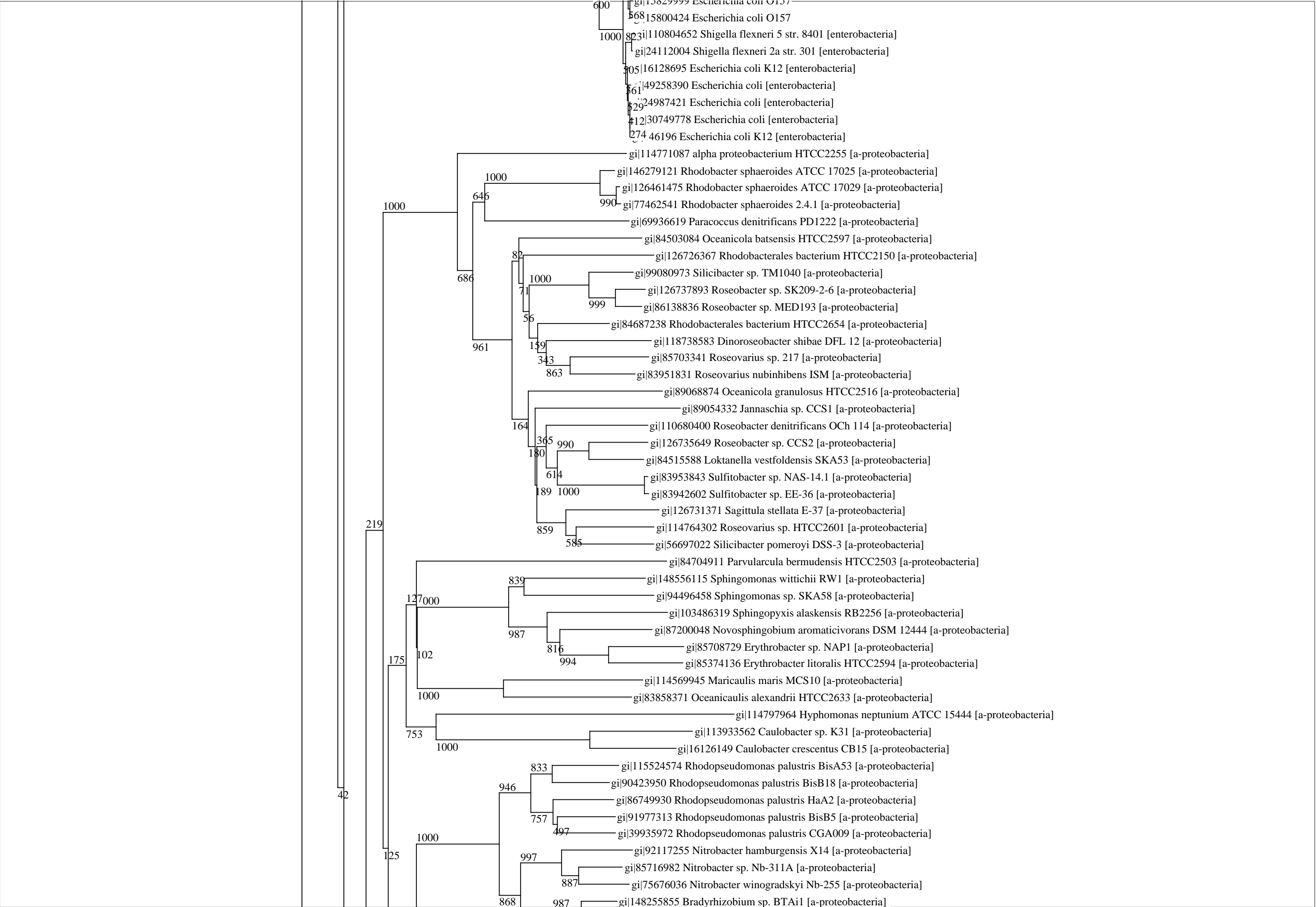

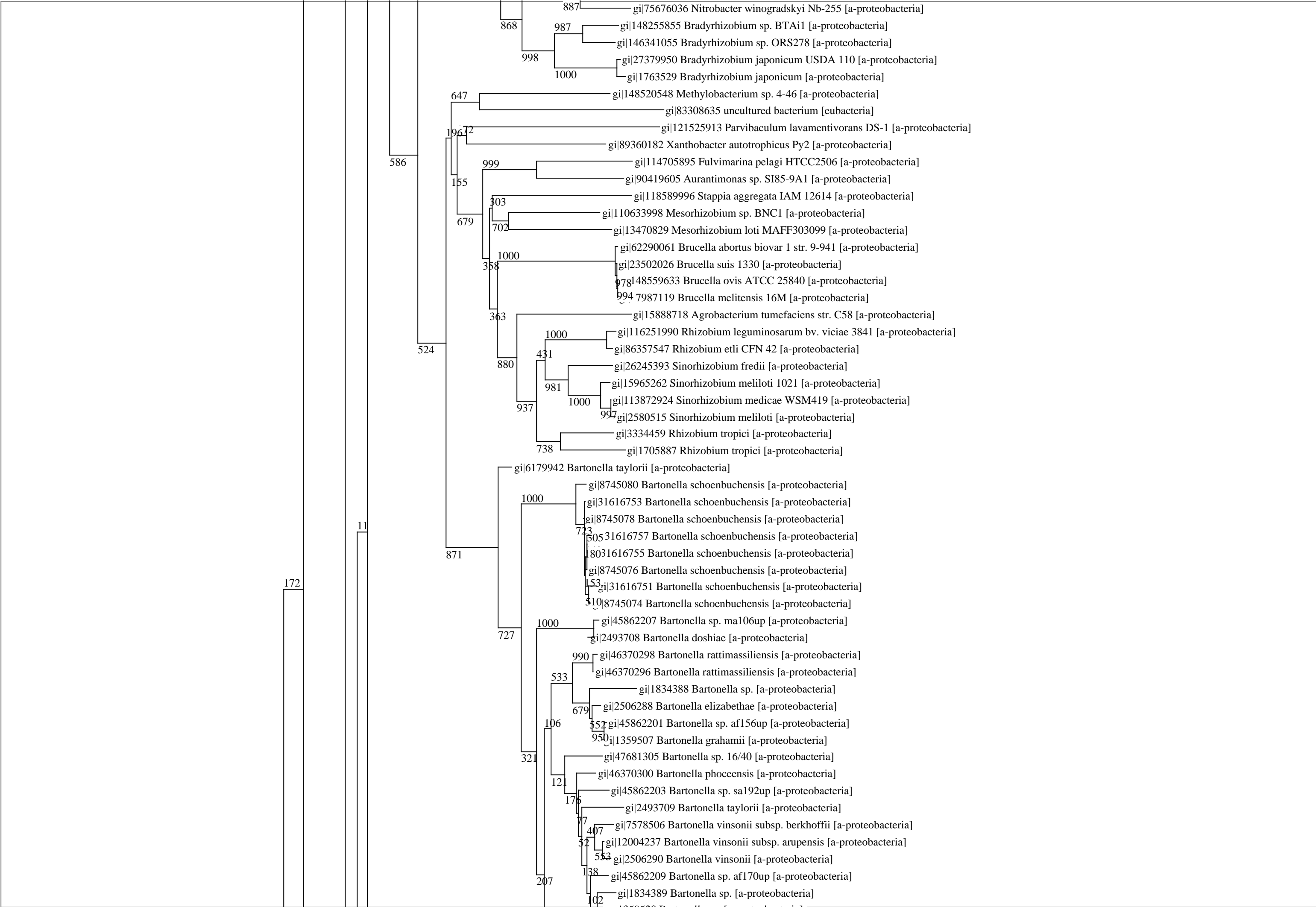

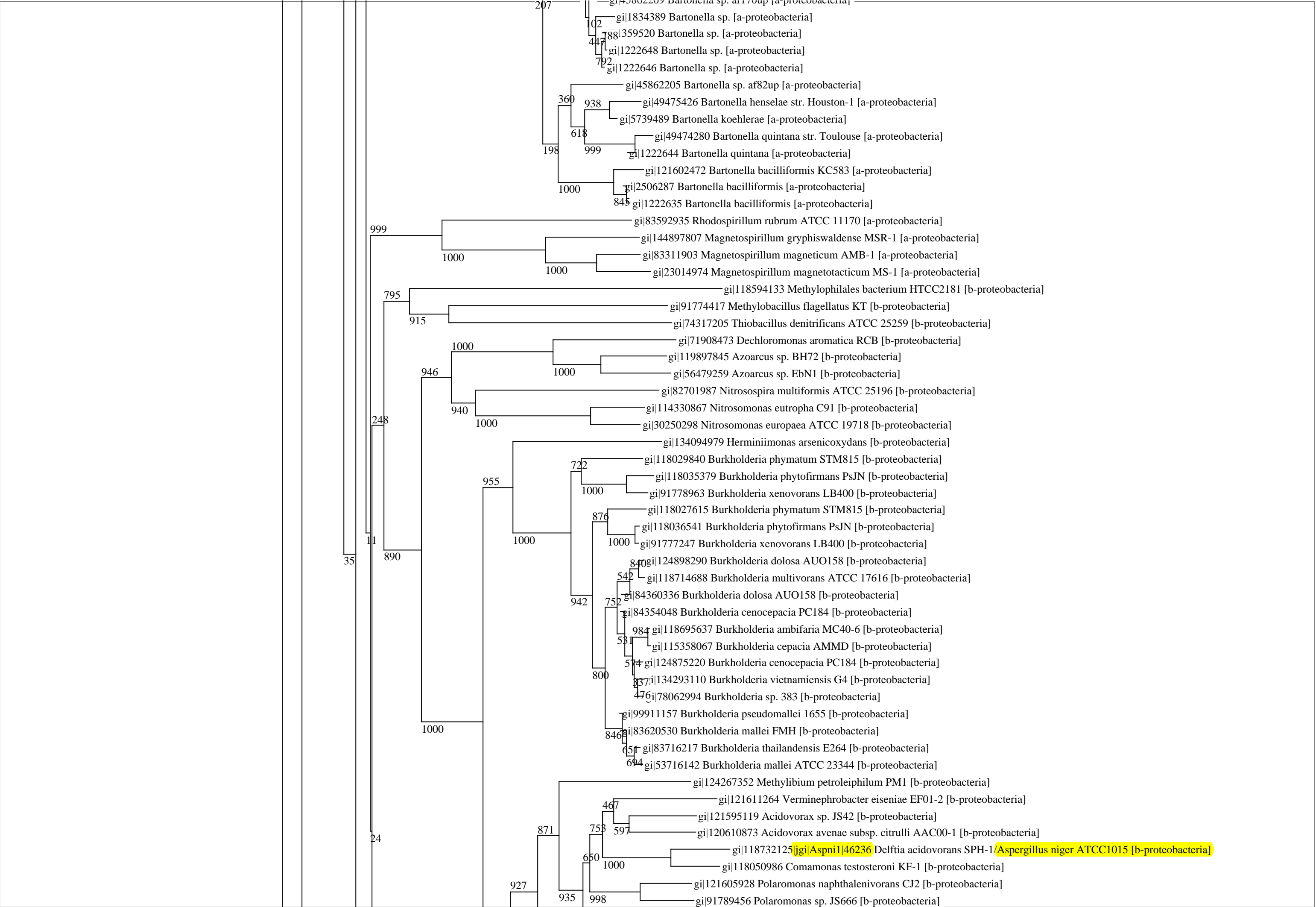

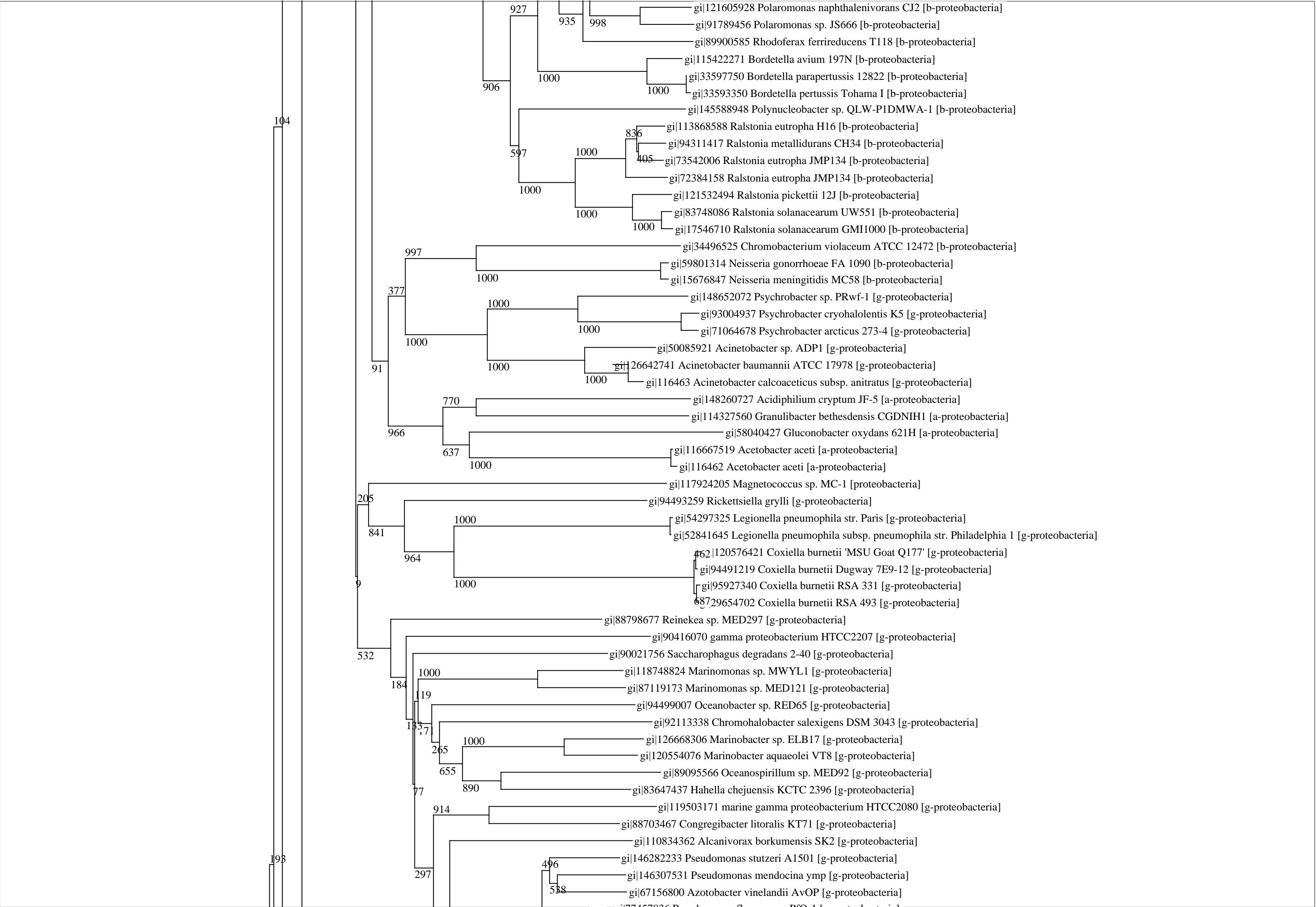

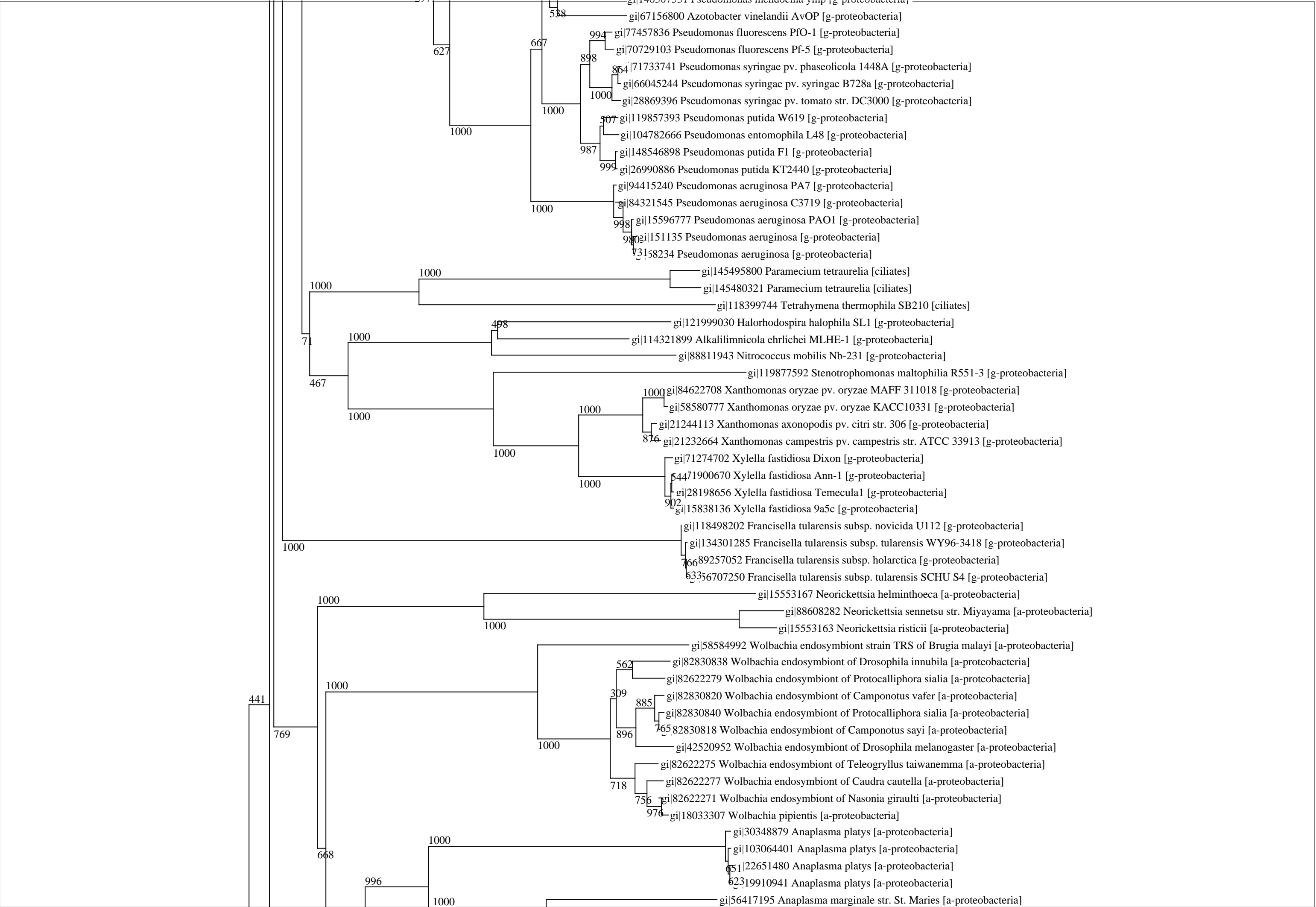

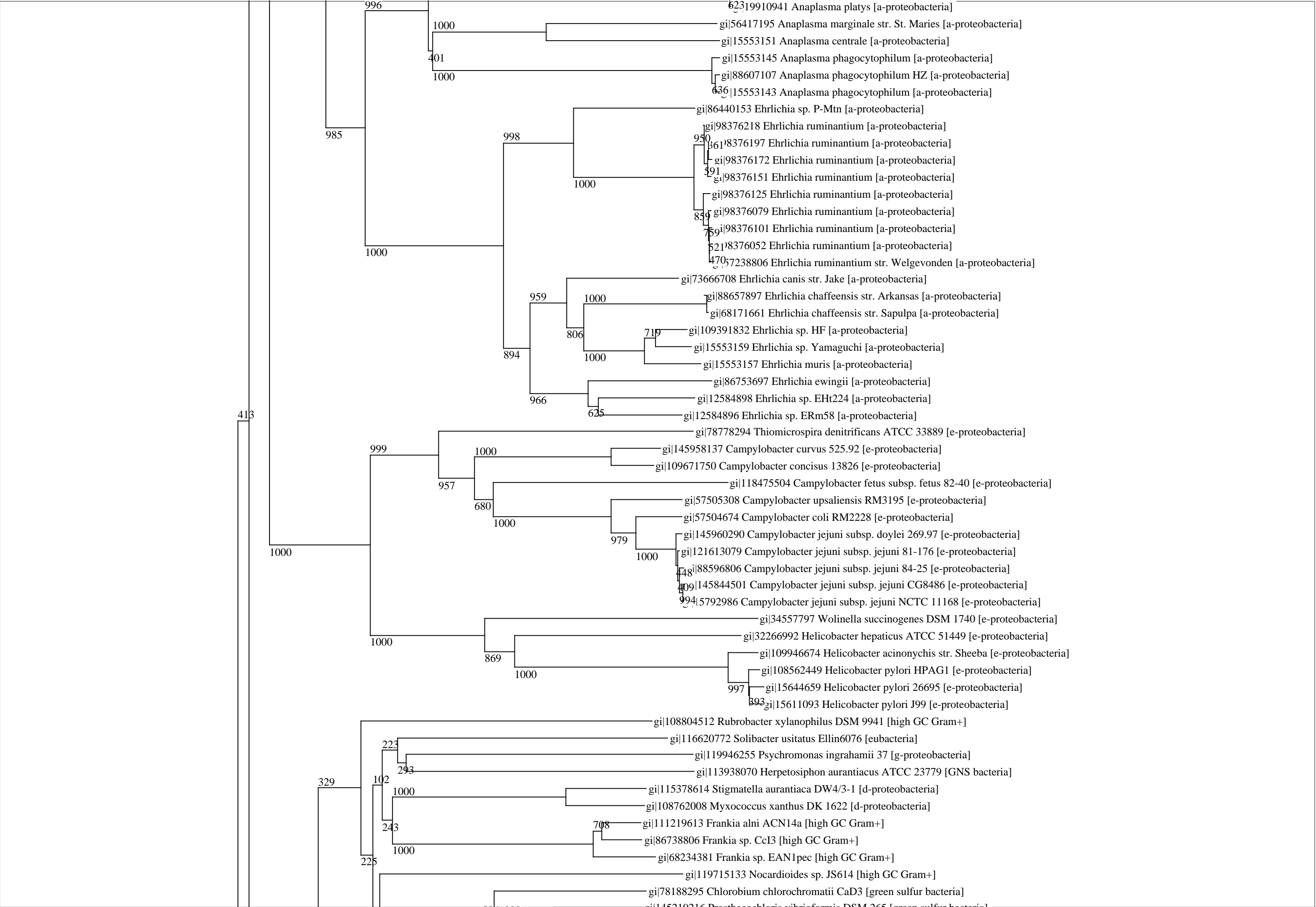

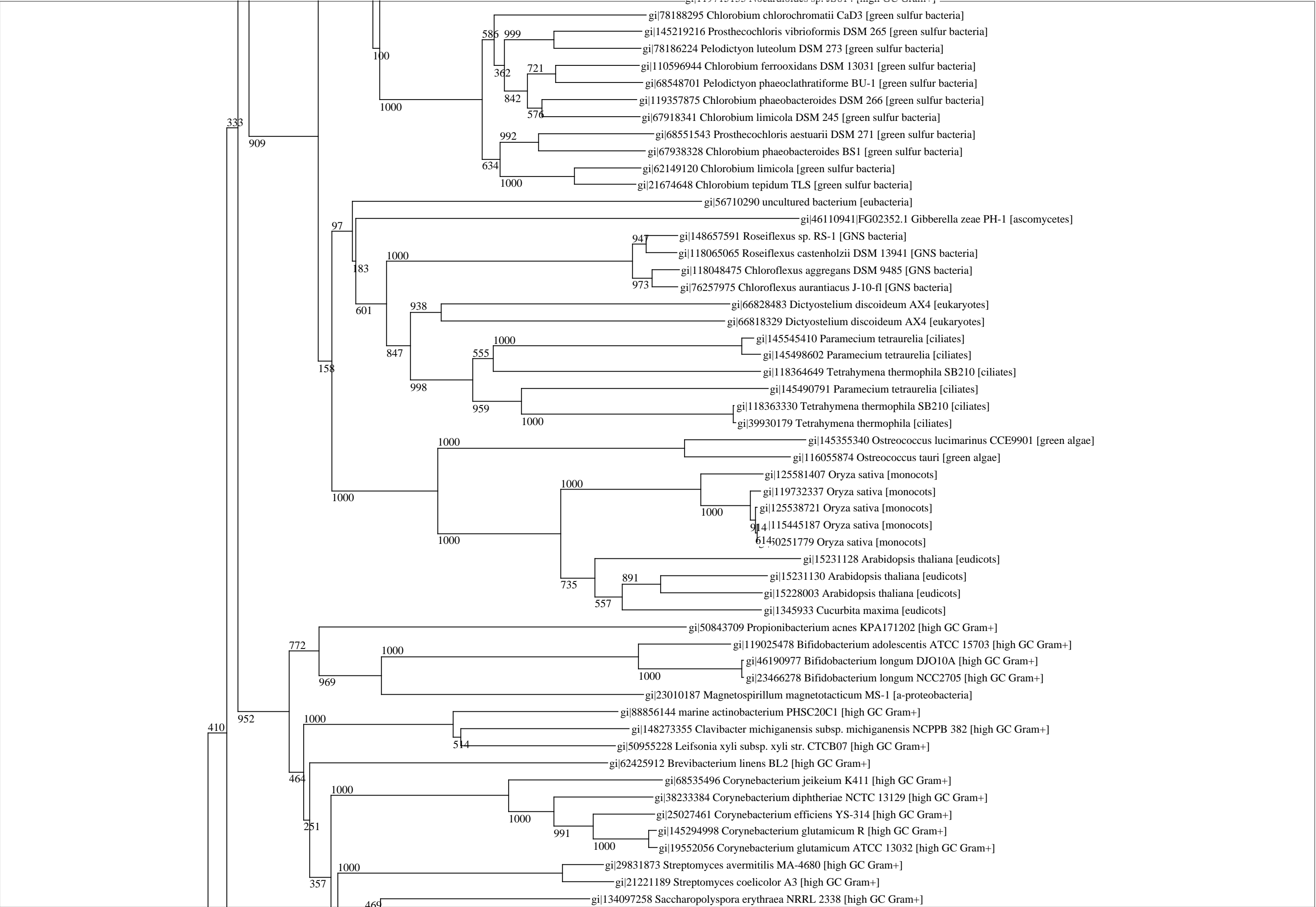

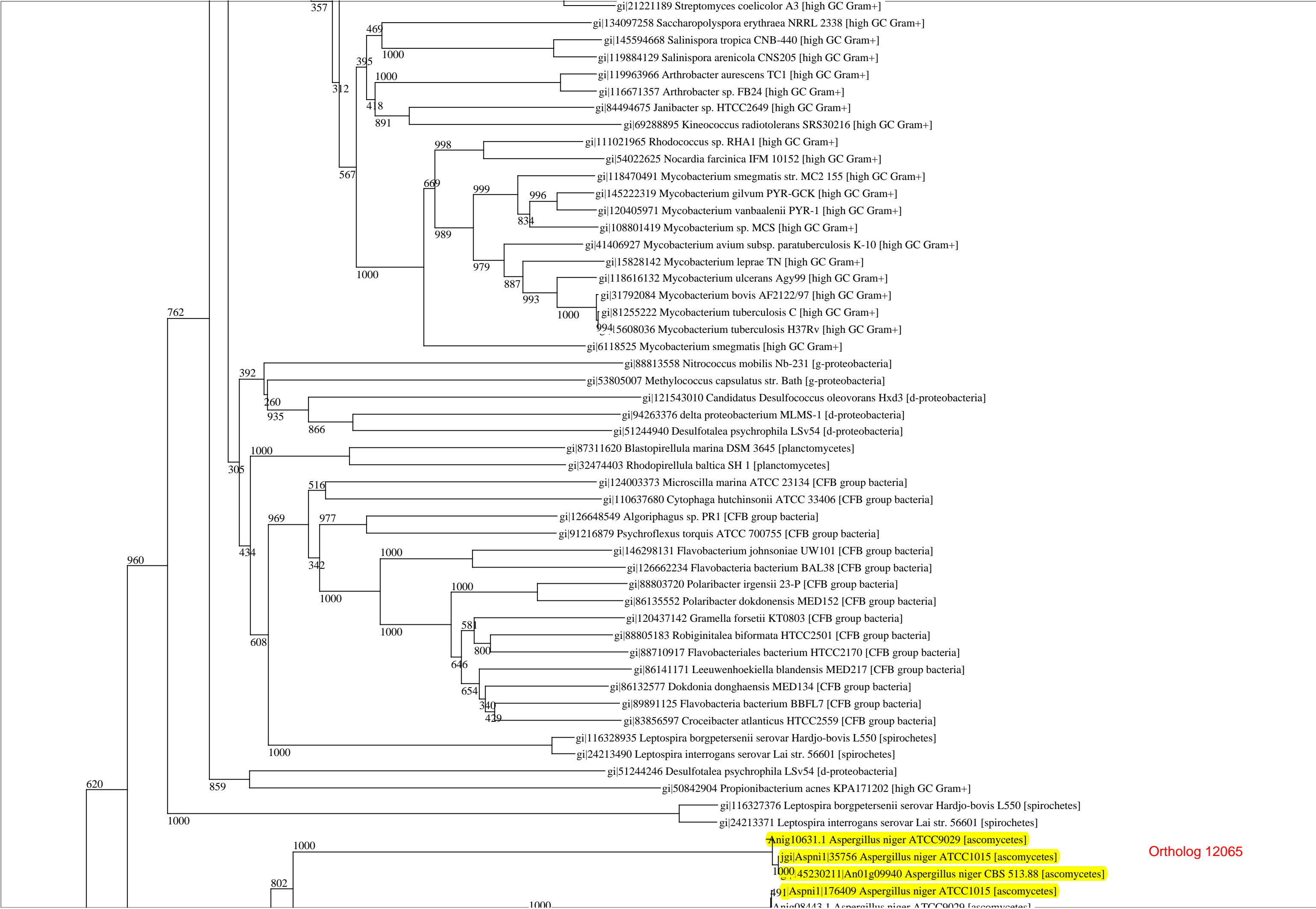

Ortholog 12065



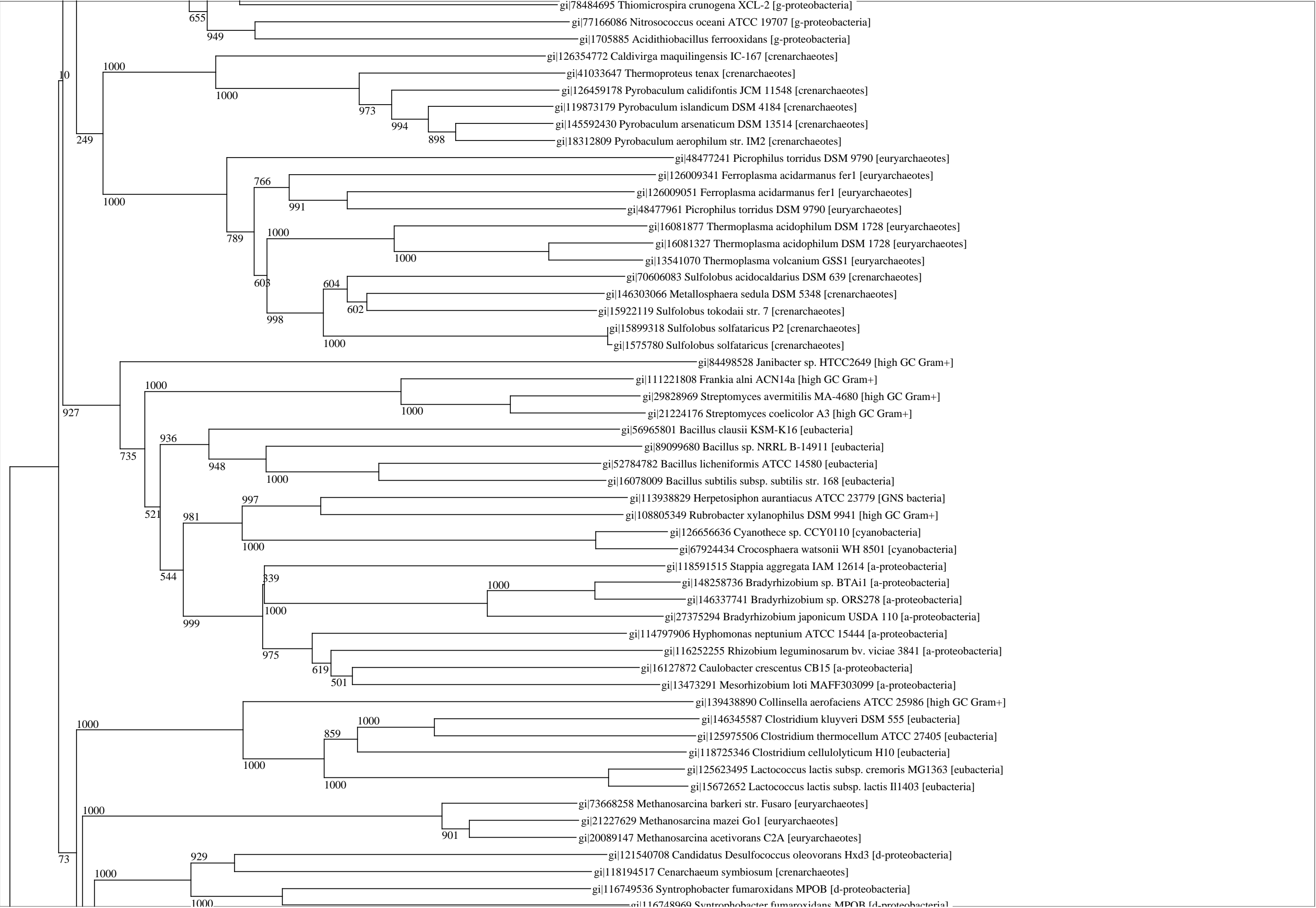

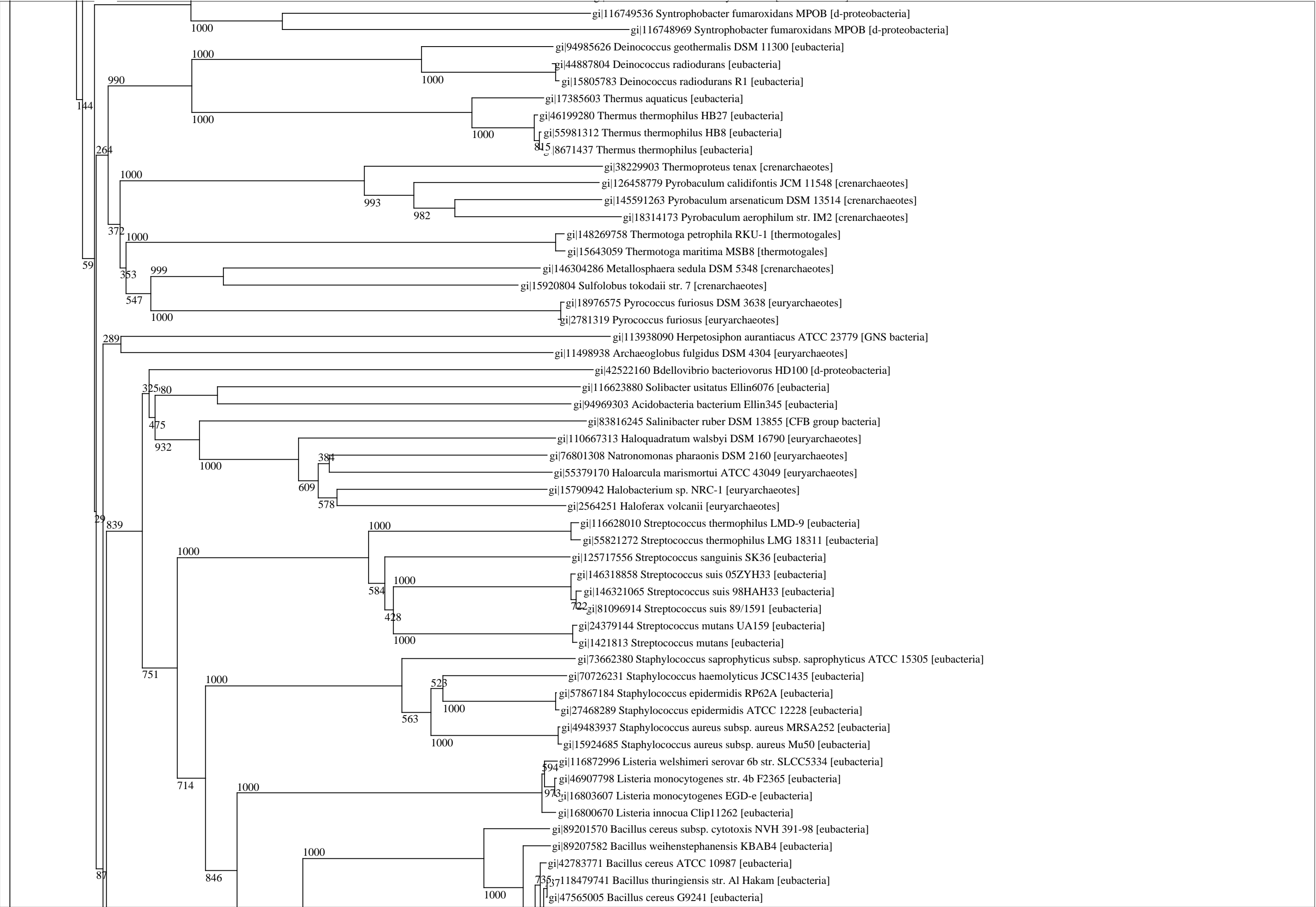

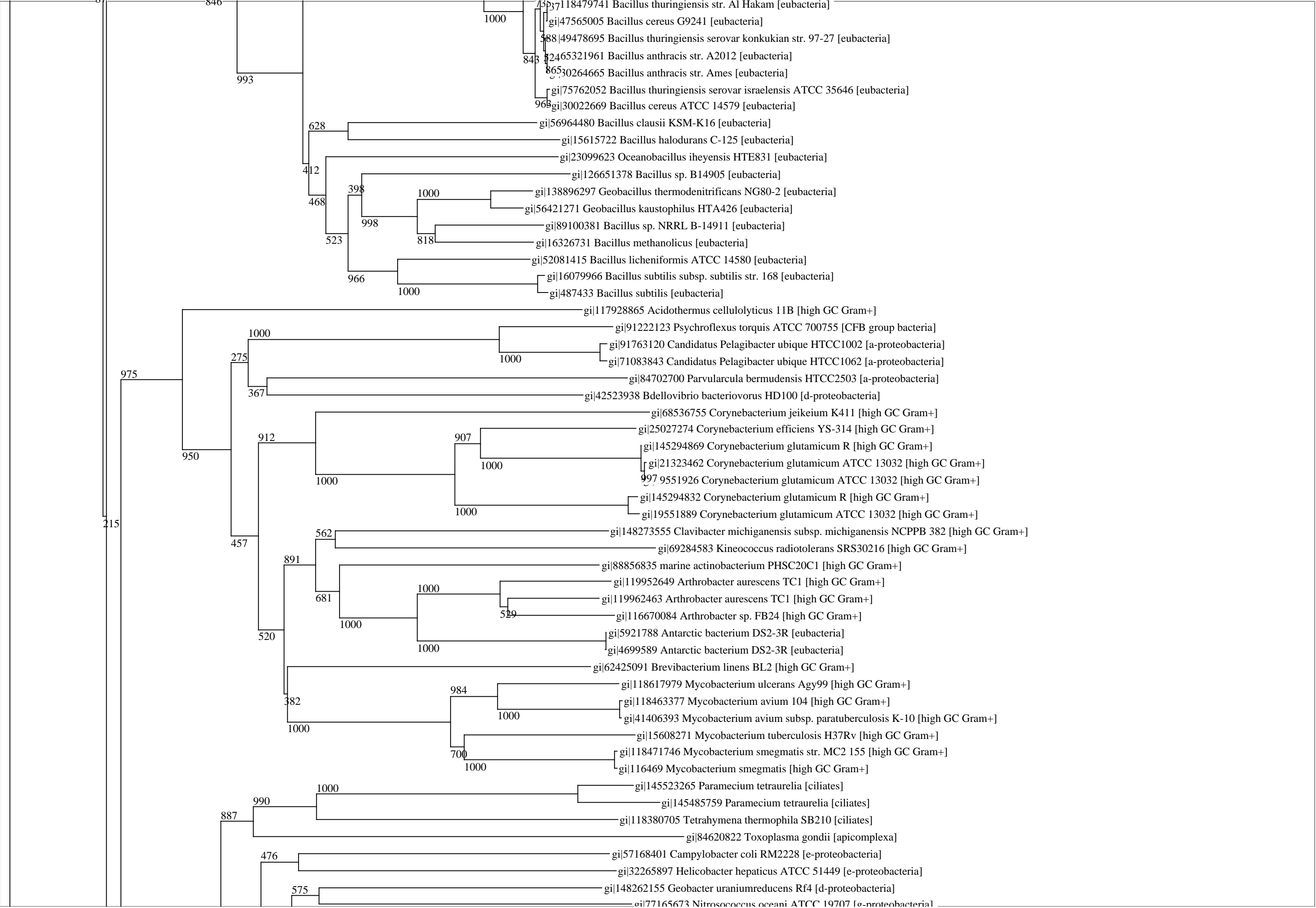

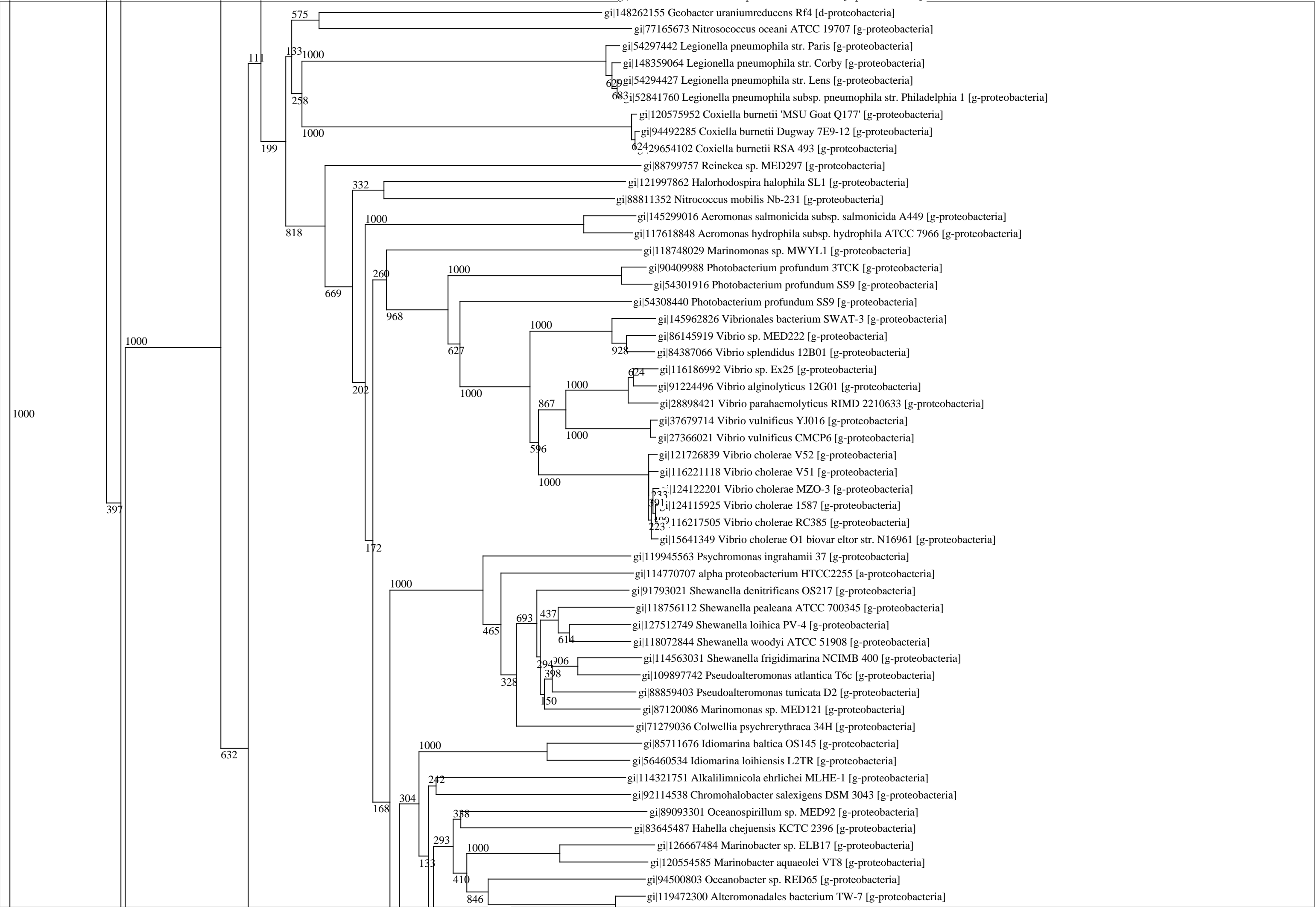

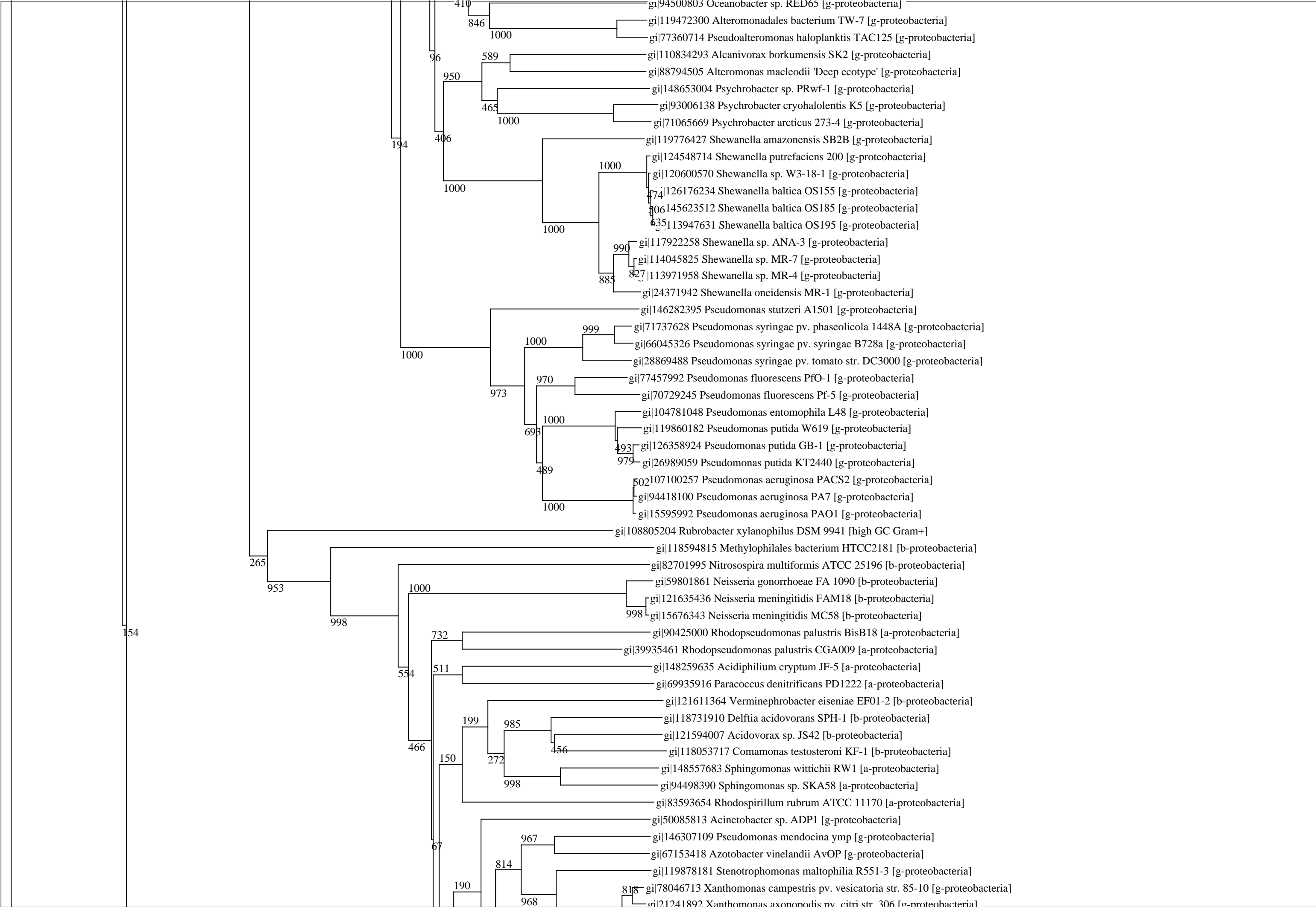

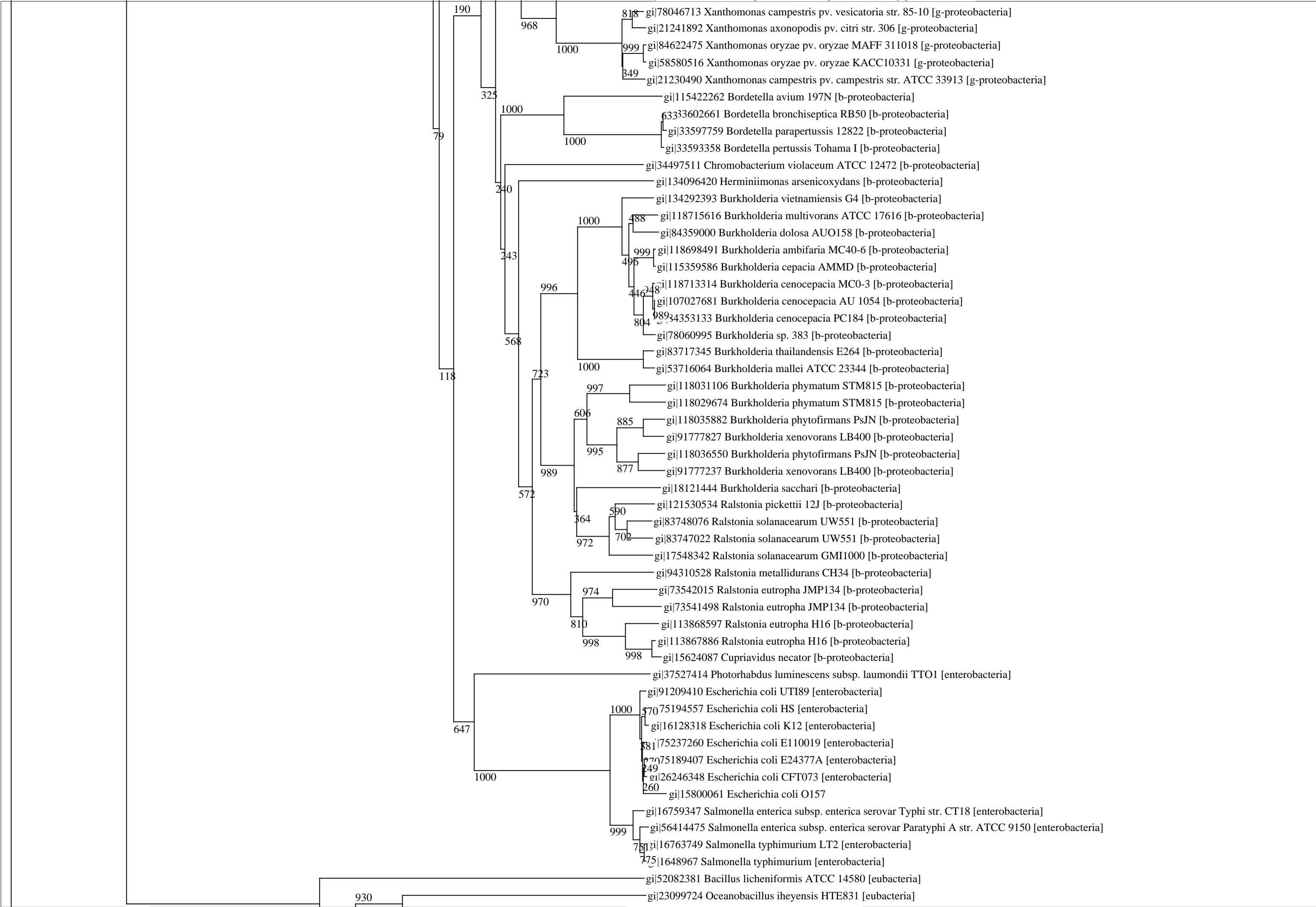

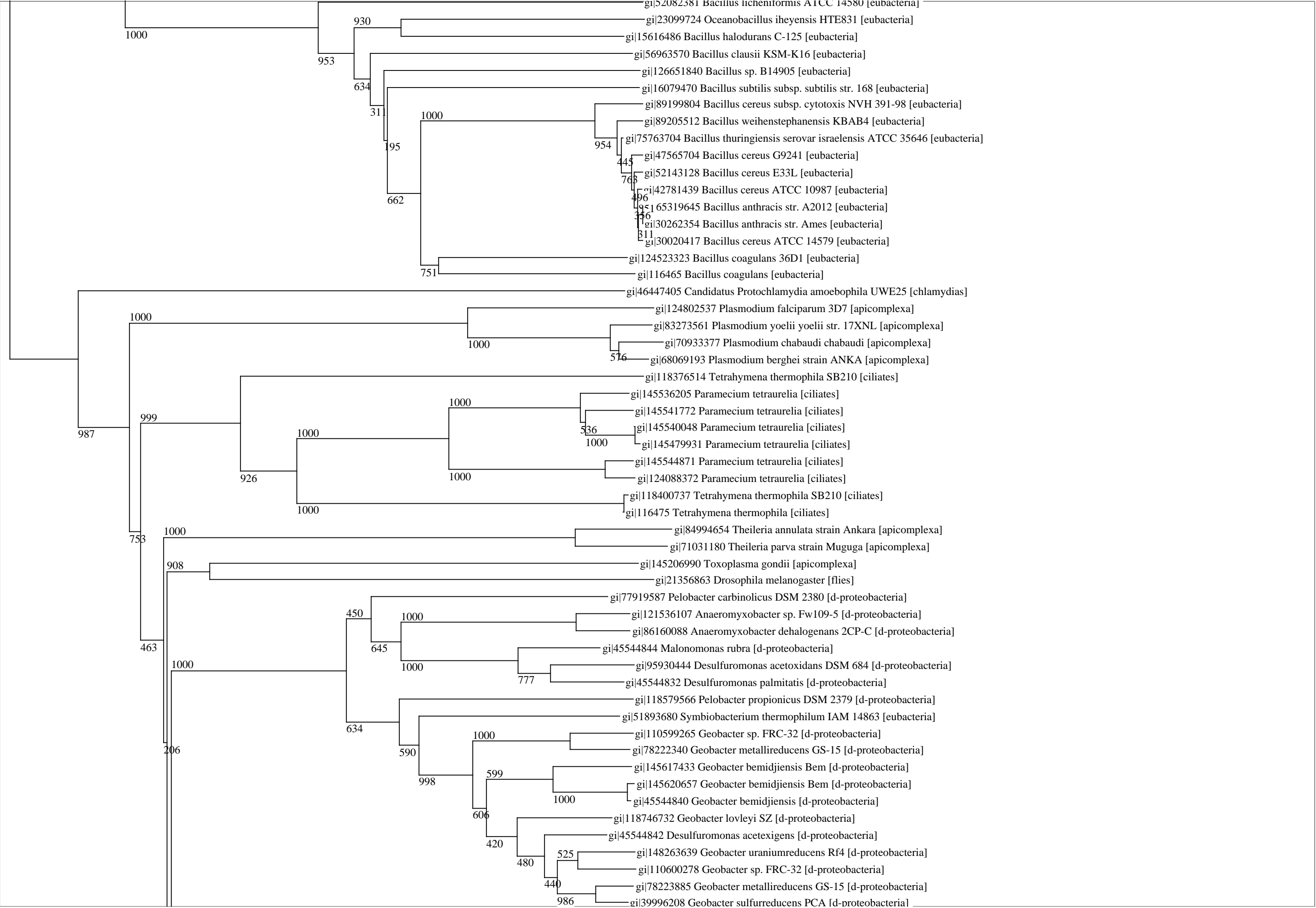

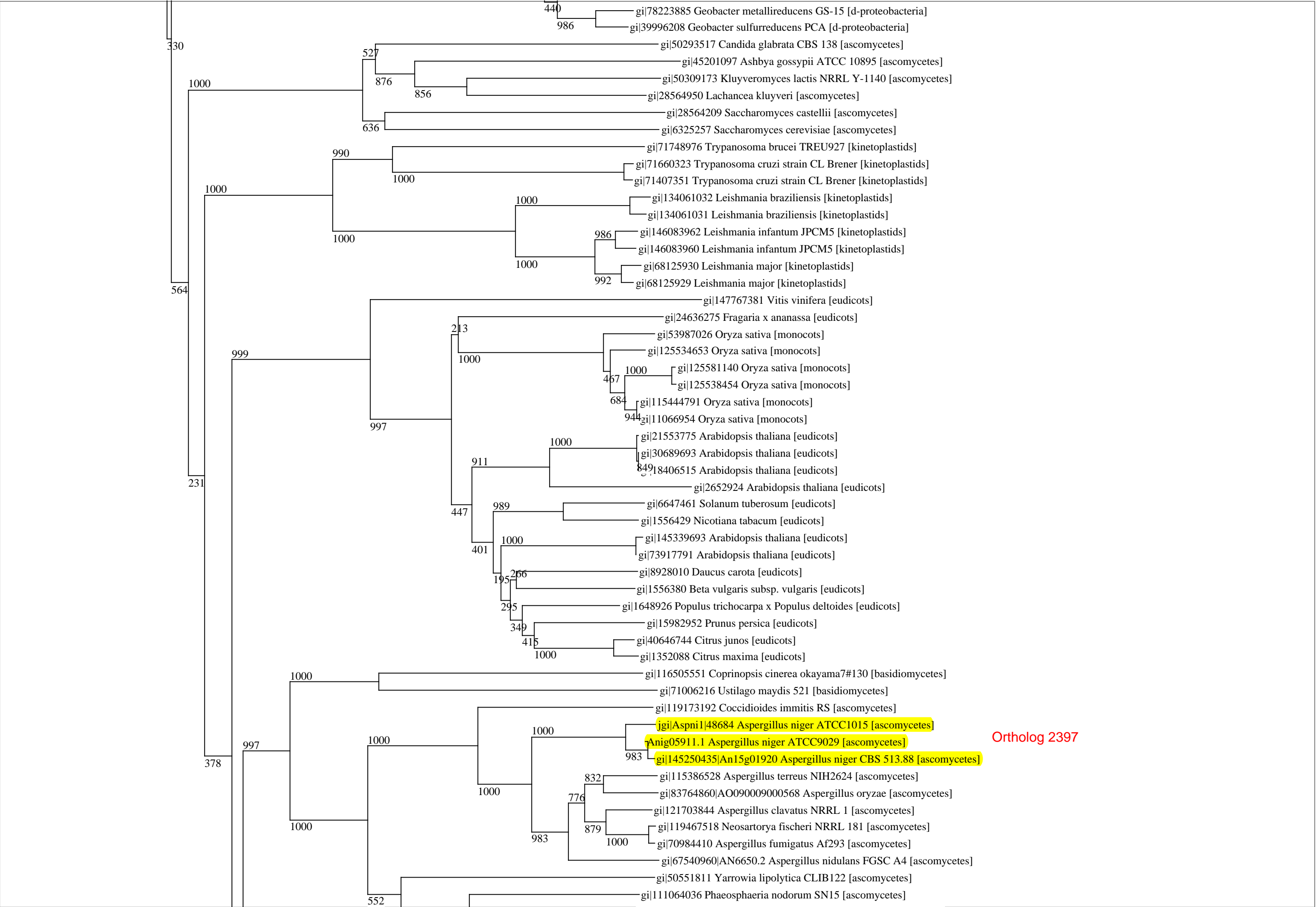

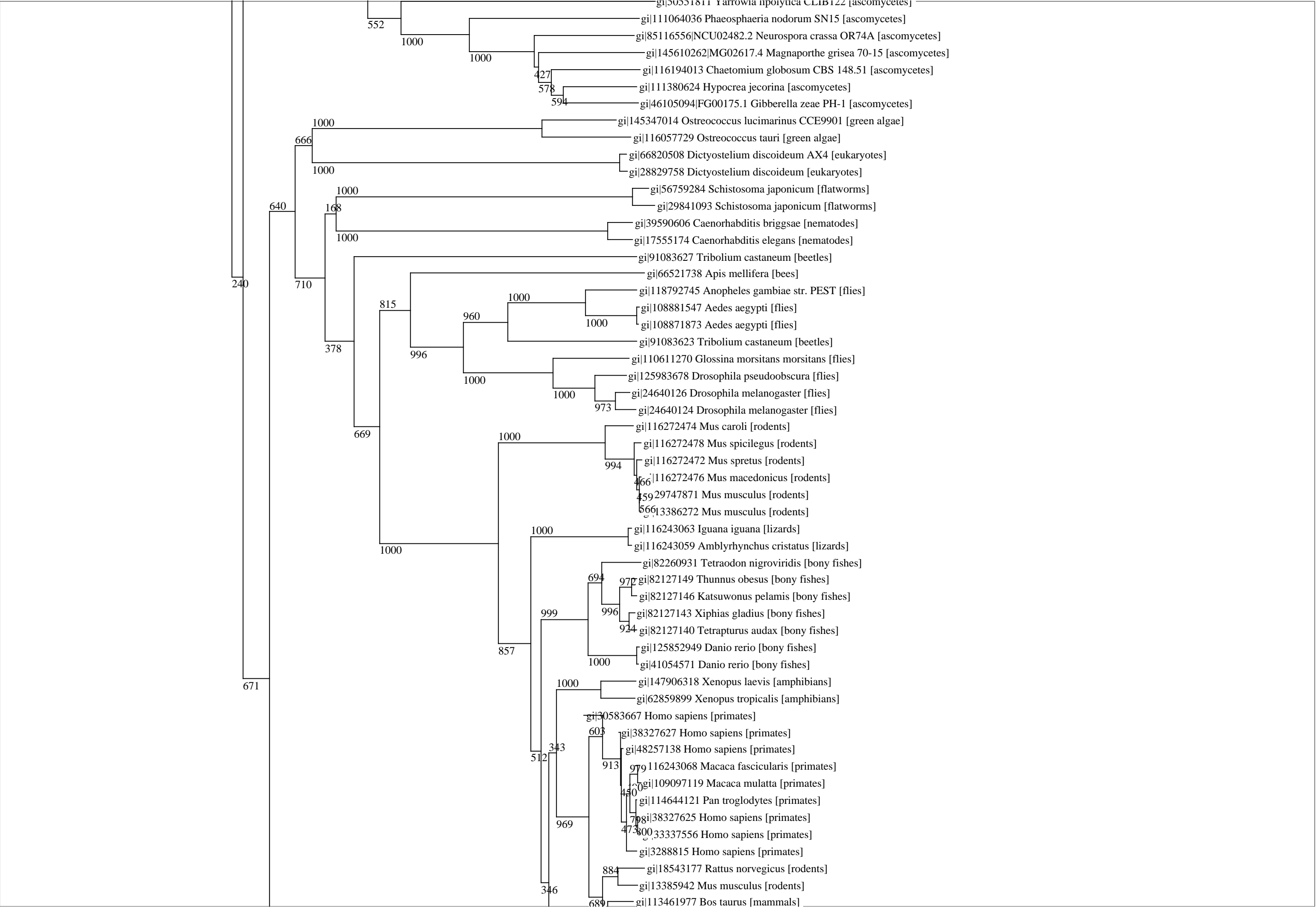

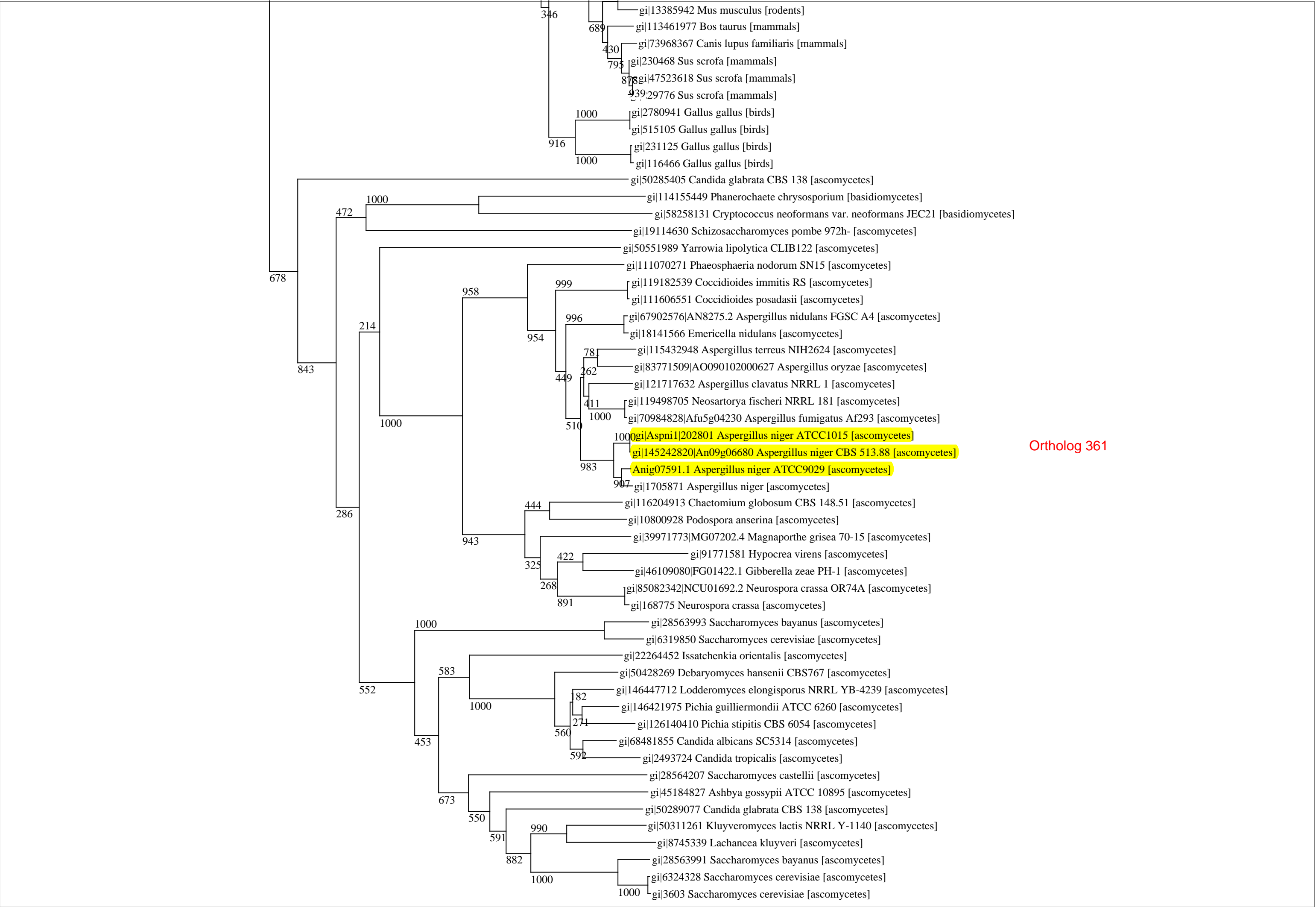

Ortholog 361

Supplement: Additional data file 13 — Phylogenetic analysis of citric acid synthases (CS), where 1,123 sequences from the NCBI nr protein database homologous to any of the six CSs of A. niger (cutoff E-value 1E-20, partial sequence ignored) were aligned together with the CSs from A. niger ATCC 9029 and ATCC 1015 to build the phylogenetic tree with 1,000 time bootstraps, using the software ClustalW. Part A is an overview while part B is the full phylogenetic tree with GI number, strain name, taxonomy, and bootstrap values. [file gb-2007-8-9-r182-S13.pdf]
