# Supplementary material for: Metabolic peculiarities of Aspergillus niger disclosed by comparative metabolic genomics
Source: Genome Biol. 2007 Sep 4;8(9):R182. doi: 10.1186/gb-2007-8-9-r182 (PMC2375020; doi:10.1186/gb-2007-8-9-r182)
Supplement: Additional data file 5 — A clickable version of Figure 2 where nodes (metabolites) are linked to the KEGG Ligand database for detailed information. [file gb-2007-8-9-r182-S5.zip › Additional data file 5/A.niger.MetNet.Metabolites.html]

Metablic network of Aspergillus niger 

The genome-wide metabolic network of Aspergillus niger (clickable version of Fig 2A). This picture presents the constructed metabolic network as "metabolite graph" with 2349 metabolites as nodes. The link between nodes is the reaction. The size of nodes is proportional to the number of reactions from or to this metabolite. The nodes are clickable (compatible to Microsoft Internet Explorer) to redirect to the corresponding entry
of the LIGAND database of KEGG.
Questions could be issued to Dr. Jibin Sun, Tel: +49 (0)531 61815350.
  
  
