# Supplementary material for: Metabolic peculiarities of Aspergillus niger disclosed by comparative metabolic genomics
Source: Genome Biol. 2007 Sep 4;8(9):R182. doi: 10.1186/gb-2007-8-9-r182 (PMC2375020; doi:10.1186/gb-2007-8-9-r182)
Supplement: Additional data file 6 — A clickable version of Additional data file 4 where nodes (reactions) are linked to the KEGG Ligand database for detailed information. [file gb-2007-8-9-r182-S6.zip › Additional data file 6/A.niger.MetNet.Reaction.html]

Metablic network of Aspergillus niger 

The genome-wide metabolic network of Aspergillus niger. This picture presents the reconstructed metabolic network as "reaction graph" with 3443 reactions as nodes. The link between nodes is the common metabolite connecting two reactions. The nodes are clickable (compatible with Microsoft Internet Explorer) to redirect to the corresponding entry of the LIGAND database of KEGG. Questions could be issued to Dr. Jibin Sun, Tel: +49 (0)531 61815350.
  
  
