# Supplementary material for: Metabolic peculiarities of Aspergillus niger disclosed by comparative metabolic genomics
Source: Genome Biol. 2007 Sep 4;8(9):R182. doi: 10.1186/gb-2007-8-9-r182 (PMC2375020; doi:10.1186/gb-2007-8-9-r182)
Supplement: Additional data file 8 — Results of a comparative assessment of the central metabolic network. [file gb-2007-8-9-r182-S8.doc]

***A comparative assessment of the central metabolic network.*** The metabolic network reconstructed from the genomic data was compared to the network of the central carbon metabolism of *A. niger* reconstructed by David et al. [17]. The network of David et al. [17] was mainly based on literature data of *A. niger* and the genomic information of *A. nidulans* and other fungi. It contains 335 reactions, 284 metabolites and 129 EC numbers. In general there is a good agreement between these two metabolic networks regarding the central metabolism. Only 14 ECs in the metabolic network of David et al. [17] could not be found in the genome-wide network reconstructed by this work. These discrepancies are summarized in Table SW2-1 (column 1). To avoid annotation errors, we tried to extract the sequences of proteins having these EC numbers from the public databases such as UniProt/Swiss-Prot ([http://www.expasy.org/enzyme](http://www.expasy.org/enzyme/)), BRENDA ([http://www.brenda.uni-koeln.de](http://www.brenda.uni-koeln.de/)) and KEGG LIGAND ([http://www.genome.ad.jp](http://www.genome.ad.jp/)) (Table SW2-1 column 3). 10 of the 14 ECs were found to have known protein sequences which were subsequently queried in the genomic database of three *A. niger* strains by sequence alignment. Proteins of six ECs have no significant homologues in *A.niger*, reinforcing the conclusion for the absence of these proteins in the genome of *A. niger*. Weak homologues (identity lower than 35%) were found for the other four ECs. However, all these homologues were confidently annotated as other functions. Clearly, most of these differential ECs belong to enzymes not well characterized so far, since merely a few or even no sequences corresponding to these ECs are known untill now (Table SW2-1). These enzymes with EC numbers inferred from literature data could give some hints to improve the functional annotation. On the other hand, the possible differences between subspecies or different species should not be neglected during the reconstruction of metabolic network based on literature data, as previously showed for 6 different *E. coli* strains [39]. The metabolic model based on literature data by David et al [17] obviously did not consider the metabolic difference between subspecies. The possibility of over-integration of information from other sequenced and closely-related genomes could also not be ruled out.

Table SW2-1. List of enzymes not found in the genomic annotation but described in the literature (David et al. 2003)

| EC | Description | Sequences available from UniProt/SwissProt | Genome database of *A*. *niger* ATCC 9029 and CBS 513.88 |
| --- | --- | --- | --- |
| 1.1.1.6 | glycerol dehydrogenase | 5 | no hits |
| 1.1.1.48 | galactose 1-dehydrogenase | 1 | no hits |
| 2.3.1.8 | phosphate acetyltransferase | 32 | no hits |
| 3.1.3.9 | glucose-6-phosphatase | 6 | no hits |
| 4.1.1.32 | phosphoenolpyruvate carboxykinase (GTP) | 52 | no hits |
| 4.2.99.11 | methylglyoxal synthase | > 50 | no hits |
| 1.1.1.60 | 2-hydroxy-3-oxopropionate reductase | 3 | weak homologs |
| 2.7.1.31 | glycerate kinase | 9 | weak homologs |
| 1.2.1.22 | lactaldehyde dehydrogenase | 1 | weak homologs |
| 4.2.1.39 | gluconate dehydratase | 2 from TrEMBL | weak homologs |
| 1.1.1.78 | methylglyoxal reductase (NADH) | no |  |
| 3.1.3.22 | mannitol-1-phosphate-phosphatase | no |  |
| 3.5.4.17 | adenosine-phosphate deaminase | no |  |
| 4.1.2.2 | erythrose-1-phosphate synthase | no |  |
