# Supplementary material for: Metabolic peculiarities of Aspergillus niger disclosed by comparative metabolic genomics
Source: Genome Biol. 2007 Sep 4;8(9):R182. doi: 10.1186/gb-2007-8-9-r182 (PMC2375020; doi:10.1186/gb-2007-8-9-r182)
Supplement: Additional data file 11 — Phylogenetic analysis of the S-adenosylmethionine tRNA ribosyltransferase. [file gb-2007-8-9-r182-S11.doc]

**Phylogenetic analysis of the enzyme S-adenosylmethionine tRNA ribosyltransferase unique to *A. niger***

S-adenosylmethionine tRNA ribosyltransferase (gene name queA) catalyzes the penultimate step in the biosynthesis of the tRNA wobble nucleoside queuosine. This enzyme isomerizes the ribosyl moiety of the cofactor S-adenosylmethionine (AdoMet) and attaches it to a modified-tRNA precursor (tRNA bound 7-(aminomethyl)-7-deazaguanine (preQ1)) [31,32]. Queuosine (Q) is one of the most complex modifications occurring at the wobble position of tRNAs with GUN anticodons specifying the amino acids asparagine, aspartate, histidine, and tyrosine. Queuosine is found in nearly all eukaryotic and eubacterial organisms, with the known exceptions of *Saccharomyces cerevisiae* and *Mycoplasma* spp. [53]. It was known that Q is synthesized *de novo* exclusively in eubacteria; for many eucaryotes the compound is usually an essential nutrient [31,32]. According to InterPro (a database of protein families, domains and functional sites, <http://www.ebi.ac.uk/interpro>), 594 proteins are found to have the signature of IPR003699 (signature of queA), almost all of them belonging to Bacteria (4 exceptions, status of June 5^th^ 2007).

The biosynthesis pathway for queuosine in *E. coli* is composed of five steps [54]. The first four steps are already elucidated and associated with genes, queC, queF, tgt and queA, respectively. The gene corresponding to the final step is still not known. *A.niger* is found to have homolog to tgt and queA, but not to queC and queF. Although only some of the enzymes of the Q biosynthesis pathway are found in *A. niger*, this organism can grow without supply of Q as nutrient [55], indicating it may possess another pathway for *de novo* biosynthesis of Q. It is less possible that *A. niger* doesn’t require Q at all, like *S. cerevisiae*, since it has several genes involved in the Q metabolism.

A phylogenetic analysis was performed for the enzyme QueA, based on the multiple sequence alignment (Figure AF5-1). Besides another two genes from two fungal species, *Aspergillus terreus* and *Chaetomium globosum*, the next closest homolog is from *Salmonella typhimurium*. Especially, their N-termini (145-340 aa) have identities as high as 40%. The other closer homologs are from Bacillales, Lactobacillales, Clostridiales and Gammaproteobacteria.


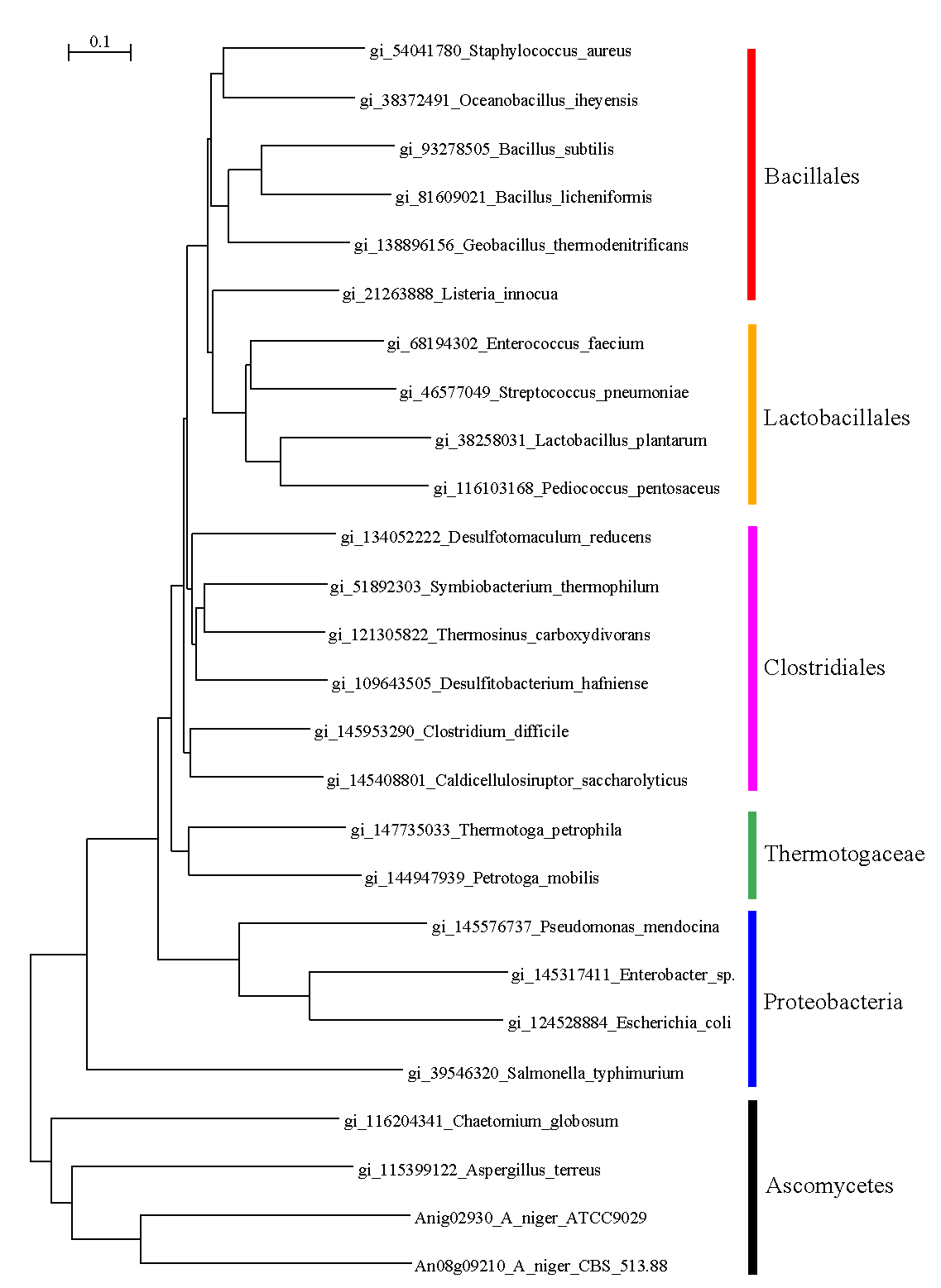


Fig. AF5-1. Phylogenetic analysis of the homologs to the protein An08g09210 (S-adenosylmethionine tRNA ribosyltransferase) of *A. niger*. The amino acid sequence of An08g09210 is queried to the non-redundant protein database of NCBI (nr) via NCBI blast server. 597 non-redundant hits were found at E-value cutoff of 1e-10. Here shows only the analysis of representative sequences with E-value cutoff of 1e-50.
